# Predicting RNA splicing from DNA sequence using Pangolin

**DOI:** 10.1186/s13059-022-02664-4

**Published:** 2022-04-21

**Authors:** Tony Zeng, Yang I Li

**Affiliations:** 1 The College, University of Chicago, Chicago, 60637 IL USA; 2 Section of Genetic Medicine, Department of Medicine, University of Chicago, Chicago, 60637 IL USA

## Abstract

Recent progress in deep learning has greatly improved the prediction of RNA splicing from DNA sequence. Here, we present Pangolin, a deep learning model to predict splice site strength in multiple tissues. Pangolin outperforms state-of-the-art methods for predicting RNA splicing on a variety of prediction tasks. Pangolin improves prediction of the impact of genetic variants on RNA splicing, including common, rare, and lineage-specific genetic variation. In addition, Pangolin identifies loss-of-function mutations with high accuracy and recall, particularly for mutations that are not missense or nonsense, demonstrating remarkable potential for identifying pathogenic variants.

**Supplementary Information:**

The online version contains supplementary material available at (10.1186/s13059-022-02664-4).

RNA splicing is an intricate gene regulatory mechanism that removes introns from pre-mRNAs. How the cell chooses which splice sites are used during RNA splicing remains unclear, but it is well known that DNA sequence is a key determinant of splice site usage [4, 26, 29]. Many studies have now shown that both rare and common genetic variants contribute to human disease by disrupting RNA splicing [1, 22]. Thus, predicting RNA splicing from DNA sequences can greatly aid the identification and interpretation of disease-causing mutations.

Due to the complexity of the sequence determinants of RNA splicing, end-to-end deep neural networks are well-suited to learn features directly from DNA sequence to predict splicing outcomes of interest. Examples of state-of-the-art deep neural networks for predicting RNA splicing from DNA sequence include MMSplice [7] and SpliceAI [14], both of which have been used successfully to predict pathogenic mutations that impact splicing. Nevertheless, both methods have limitations. For example, MMSplice predicts usage of cassette exons rather than that of splice sites, and thus is likely to overlook disease-causing mutations that disrupt complex splicing patterns. Furthermore, neither MMSplice nor SpliceAI predicts tissue-specific splicing (though a newer version of MMSplice, MTSplice [6] can predict tissue-specific splicing). Several methods that do not use deep learning have also been successfully used to study sequence determinants of splicing, including HAL [25] and MaxEntScan [30], which use an additive linear model and a maximum entropy model respectively. However, deep learning based approaches have been shown to outperform these methods in a variety of prediction tasks [7, 14].

To model splicing in a quantitative, tissue-specific manner, we developed Pangolin, a deep neural network that predicts splicing in four tissues—heart, liver, brain, and testis—which represent some of the major mammalian organs (Fig. 1a). This is an improvement over SpliceAI and MMSplice, which produce the same prediction for any tissue. In addition, Pangolin can predict the usage of a splice site in addition to the probability that it is spliced (Fig. 1a). This is an improvement over SpliceAI, which merely reports predictions for whether a dinucleotide is a splice site or not [14] (Additional file 1: Supplementary Note 1). Pangolin’s model architecture consists primarily of 16 stacked residual blocks with skip connections, each of which contains batch normalization, ReLU activation, and convolutional layers (“Methods” section). Pangolin’s architecture resembles that used in SpliceAI, which allows modeling of features from up to 5000 base pairs upstream and downstream each target splice site. An important difference is the addition of multiple outputs to the final neural network layer of Pangolin, allowing prediction of splice site usage across different tissues.

**Fig. 1:**
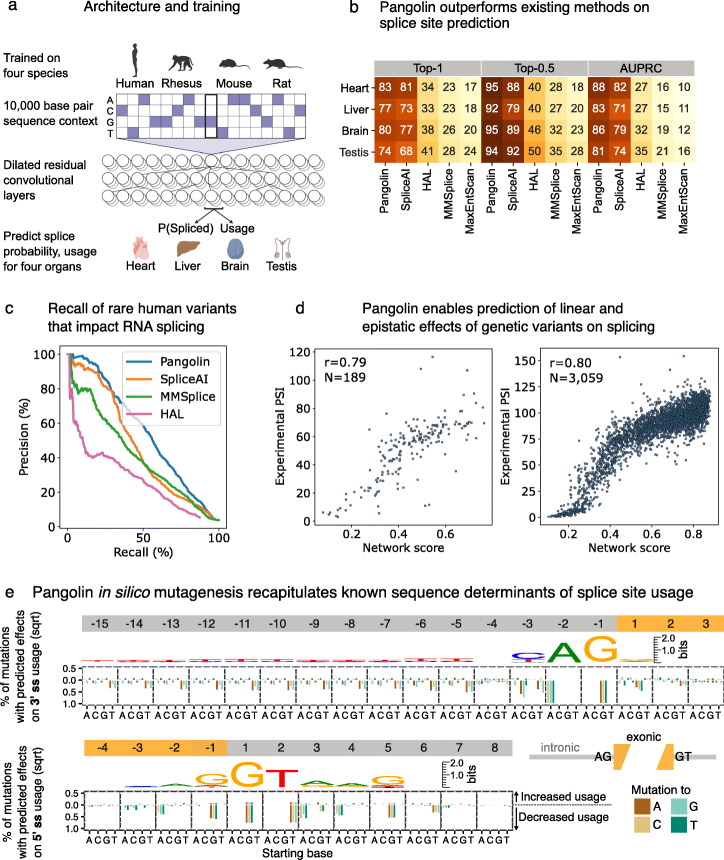
Overview of Pangolin and evaluation. **a** Schematic and architecture of Pangolin. **b** Heatmap summarizing the performance of Pangolin, SpliceAI, HAL, MMSplice, and MaxEntScan with respect to three metrics including top-1 accuracy. **c** Precision-recall curves representing the precision and recall from multiple methods for the prediction of splice-disrupting variants as identified in Cheung et al. [8] (1050 splice-disrupting variants out of 27,733 total). **d** Scatter plots showing measured versus predicted effects of single genetic variants (left) or a combination of genetic variants (right) on RNA splicing. Measured effects of single genetic variants and combinations of variants were obtained from Julien et al. [15] and Baeza-Centurion et al. [3] respectively. **e** In silico mutagenesis of 6416 exons from human chromosomes 7 and 8. Barplots show for each base the percent of mutations (square root) predicted to increase or decrease usage by at least 0.2

To train Pangolin, we used data from four species—human, rhesus macaque, rat, and mouse—as we reasoned that prediction of splice site usage in multiple tissues may require a larger training set compared to that used in SpliceAI and MMSplice, which were trained using human sequences only. Using sequences from multiple species [18] and quantitative over binary labels [2, 19] have been shown to substantially improve prediction in some applications but have not yet been tested, to the best of our knowledge, in the context of RNA splicing. Specifically, we processed sequence and RNA splicing measurements from the four aforementioned species (Fig. 1a, Additional file 2: Table S1), by using SpliSER [10] to quantify the usage of all splice sites after mapping RNA-seq data from heart, liver, brain, and testis from up to 8 samples per species per tissue (“Methods” section). Next, we split genes into a training set and a test set. The training set consists of genomic positions from genes on human chromosomes 2, 4, 6, 8, 10–22, X, and Y, including splice and non-splice sites, while the test set consists of positions from genes on human chromosomes 1, 3, 5, 7, and 9. To add training sequences from rhesus macaque, rat, and mouse, we used genes that are not orthologs or paralogs of genes from the test human chromosomes (“Methods” section). This limits the possibility that our model learns human patterns of RNA splicing from orthologous sequences.

We evaluated the performance of Pangolin on predicting splice sites alongside popular methods including MaxEntScan [30], SpliceAI [14], MMSplice [7], and HAL [25]. We first compared all methods in terms of their splice site predictions on test chromosomes using the top-1 and top-0.5 metric (“Methods” section). The top-1 (resp. top-0.5) metric measures the fraction of correctly predicted splice sites at the score threshold where the number of predictions equals the total number (resp. half the number) of labeled splice sites. We also used the area under the precision-recall curve (AUPRC) to assess performance on predicting splice site locations. Across all tissues tested, Pangolin achieved an average top-1 accuracy of 79% and AUPRC of 0.85—an improvement over SpliceAI, which achieved an average top-1 accuracy of 75% and AUPRC of 0.77. Both Pangolin and SpliceAI substantially outperformed the other three tested methods—MMSplice, HAL, and MaxEntScan—which achieved average top-1 accuracies and AUPRC lower than 37% and 0.30, respectively (Fig. 1b). When considering only the top half most confident splice site predictions, Pangolin also outperformed SpliceAI (94% vs 87% top-0.5 accuracy) and other methods (Fig. 1b). In addition, we found that the improvements in Pangolin’s performance were similar for genes with low identity to training-set genes from other species, indicating that any orthologous sequences that were not filtered out from our training set only minimally affect Pangolin’s predictions (Additional file 1: Supplementary Note 2). To better understand the improved performance of Pangolin over SpliceAI, we trained multiple intermediate Pangolin models and evaluated improvements in AUPRC separately for each model. We found that (i) using splicing data from multiple tissues instead of just one, (ii) using data from multiple species instead of just human, and (iii) using quantitative measurements of splice site usage along with binary classifications all improved AUPRC (Additional file 1: Supplementary Note 3). These observations indicate that increasing the number of species and tissues used for training can further improve our model’s performance.

We next evaluated the methods in terms of their ability to predict the effects of rare variants on RNA splicing. We used data generated from a Sort-seq assay, MFASS, that tested the effects of 27,733 exonic and intronic variants from the Exome Aggregation Consortium (ExAC) on exon usage using minigene reporters [8], most of which are extremely rare in the human population. About 3.8% of tested variants were found to strongly affect exon usage (*Δ*inclusion index ≤− 0.5) and were therefore defined as splice-disrupting variants (SDVs) [8]. We tested the ability of Pangolin, SpliceAI, MMSplice, and HAL to distinguish SDVs from other variants. Pangolin achieved an AUPRC of 0.56, outperforming all other methods (Fig. 1c, “Methods” section), while SpliceAI scored the second best AUPRC (0.47). In particular, at a precision of 80%, Pangolin achieves a recall of 29%, indicating that highly confident predictions (>80% precision) from Pangolin capture a substantial fraction of rare variants that disrupt RNA splicing. Interestingly, we found that Pangolin’s performance is substantially better for variants near splice sites (AUPRC of 0.75, distance of 0-9 bases) than for farther variants (AUPRC <0.35, distance >9 bases), which may be attributable to the relative rarity of distant splice-disrupting variants (Additional file 1: Fig. S1). Further benchmarking of Pangolin, SpliceAI, and MMSplice on in vivo splicing efficiency data from a massively parallel splicing assay (MAPSy) [28] revealed that Pangolin’s predictions of genetic effects were most highly correlated with those measured using MAPSy (Pearson correlations of 0.61, 0.50, 0.37 for Pangolin, SpliceAI, and MMSplice respectively) (Additional file 1: Fig. S2, “Methods” section).

Pairwise and higher-order epistatic interactions between variants can frequently result in splicing outcomes that differ from those caused by single variants. To evaluate our ability to predict the effects of multiple mutations, we used Pangolin to predict percent-spliced-in (PSI) of exon 6 of the *FAS* gene with different single-nucleotide substitutions (*n* = 189) or with a combination of multiple substitutions (*n* = 3059) (“Methods” section). We then compared our predictions to the experimental PSIs determined using minigene reporter assays [3, 15]. Spearman correlation between predicted and experimental PSI was high for single substitutions (*r*_single_=0.79) and even higher for combinations of substitutions (*r*_multiple_=0.80) (Fig. 1d). By contrast, using a linear model of the individual variants’ PSIs [3] to predict the PSIs for combinations of variants resulted in a Spearman correlation of 0.48 (Additional file 1: Fig. S3, “Methods” section). These results demonstrate Pangolin’s ability to account for epistatic effects when making predictions.

We also tested Pangolin’s performance on predicting tissue-specific splice site usage. To do this, we calculated the difference between estimated splice site usage in each tissue and the mean usage across tissues for each site in genes on the test chromosomes, then compared these observed differences to Pangolin’s predicted differences. We limited this analysis to sites whose usage in at least one tissue differed from the mean by >0.2. Across tissues, the Spearman’s *r* coefficients between the observed and predicted tissue-specific splice site usage ranged from 0.35 to 0.50 (median of 0.43) (Additional file 1: Fig. S4). Thus, Pangolin is able to capture tissue-specific splicing effects to some extent. Although these correlations are low, we note that these are comparable to or higher than those of MTSplice [6], which produced predictions for differential exon inclusion with Spearman’s *r* coefficients that ranged from 0.09 to 0.40 (median of 0.22) (Additional file 1: Supplementary Note 4). Additionally, we find that there is highly significant differential enrichment of multiple sequence motifs near correctly predicted brain- and testis-specific splice sites relative to non-tissue-specific splice sites; though, to the best of our knowledge, the motifs identified do not correspond to the binding motifs of any well-studied splice factors (Additional file 1: Fig. S5, Supplementary Note 5). We thus conclude that predicting differential splicing across tissues from sequence alone is possible but remains a considerable challenge and requires further investigation.

Next, we applied Pangolin to a variety of prediction tasks as a demonstration of multiple potential use cases. As a first use case, we performed an in silico mutagenesis on human exons to visualize the effects of mutations on splice site usage (“Methods” section). We predicted the effects on splice site usage of all possible mutations near the 3^′^ and 5^′^ splice sites for several thousand exons, and asked about the type and fraction of base changes that increase or decrease predicted usage by at least 0.2 (“Methods” section). The mutational patterns predicted to impact splicing are highly consistent with known sequence motifs near splice sites (Fig. 1e). For example, nearly all mutations away from the AG acceptor and GT donor dinucleotides are predicted to decrease usage of the 3^′^ and 5^′^ splice sites respectively. In addition, Pangolin predicts that for a large fraction of 3^′^ splice sites, upstream T to G or T to A mutations—and to a lesser extent T to C mutations—substantially reduce 3’ splice site strength. Conversely, upstream mutations to a T—and to a lesser extent mutations to a C—increase 3^′^ splice site strength. These predictions reveal the importance of polypyrimidine tract strength for the splicing of a substantial fraction of exons [9]. Overall, we found that many fewer mutations are predicted to increase splice site usage than decrease it (Fig. 1e). This finding suggests that most (but not all) exons harbor sequences that allow for near-optimal splicing accuracy. For example, we found that at the − 3 position relative to the 3^′^ splice site, mutations away from C and T cause strong decreases in splice site usage (for another example at position − 1 relative to the 5^′^ splice site, see Additional file 1: Supplementary Note 6). This is consistent with a preference of the U2AF1 splicing factor to bind 3^′^ splice sites with C or T at the − 3 position [13, 31]. Interestingly, a mutation to C or T at the − 3 position often does not conversely increase usage, suggesting that 3^′^ splice sites with an A or G at the − 3 position may be less reliant on U2AF1 binding for splice site recognition. These examples suggest that prevalent mutational effects exist which depend on sequence context and cannot be captured by standard motifs or position weight matrices.

As a second use case, we used Pangolin to aid in the prediction of common genetic variants that impact RNA splicing. We asked Pangolin to identify single-nucleotide polymorphisms (SNPs) that impact intron excision at the top 500 most significant splicing quantitative trait loci (sQTL) identified from the DGN consortium using Leafcutter (“Methods” section). We restricted analysis to sQTLs for which the lead sQTL was at most 1 kb away from a splice site, and used Pangolin as well as SpliceAI to predict the effects on intron splice site usage for all SNPs within 1 kb of splice sites (“Methods” section). We reasoned that the *p* value of the causal sQTL SNP should be generally of similar magnitude to that of the lead sQTL SNP. Thus, we compared the sQTL *p* value of the SNP predicted to have the largest effect on splicing to the lead sQTL *p* value. We found that SNPs predicted to be causal by Pangolin have sQTL *p* values that are smaller than those predicted by SpliceAI, which in turn are much smaller than those of randomly chosen SNPs (Fig. 2a).

**Fig. 2:**
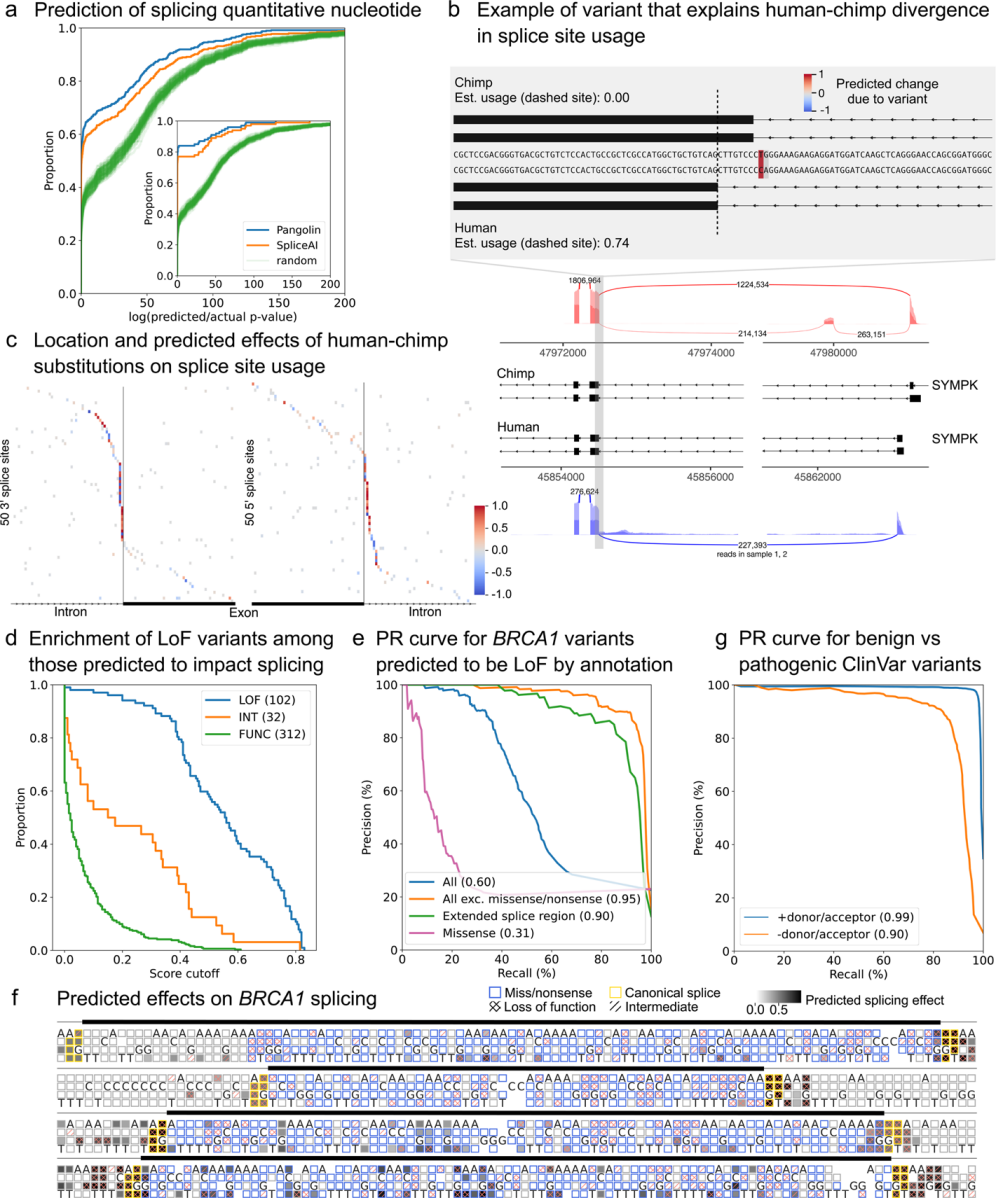
Application of Pangolin to a variety of prediction tasks. **a** Cumulative density plot of the log10 sQTL *p*-value fold difference between the SNP predicted to affect splicing and that of the lead sQTL SNP for the top 500 sQTLs identified in DGN (All predictions), or for the 100 predictions with the largest predicted effects (inset). **b** Example of a splice site that shows a large inter-species difference in usage. A single-nucleotide difference between chimp (T) and human (C) is predicted to strongly decrease (resp. increase) usage of a chimp (resp. human) splice site (dashed vertical line indicates the human site). The T (resp. C) difference likely disrupts (resp. creates) a 3’ canonical splice site in chimp (resp. human). **c** Locations and effects of SNVs ±50bp from a splice site predicted to underlie inter-species differences in splice site usage for 71 3’ and 74 5’ sites. A large fraction—but not all—of splice-altering variants are located near the canonical splice sites. **d** Survival function plots of *BRCA1* variants in splice regions as a function of their predicted effects on splicing. The variants are separated by their classification as loss-of-function (LOF, blue), intermediate effect (INT, orange), or functional (FUNC, green). We observe a huge enrichment of LOF variants among variants with large predicted splicing effects. **e** Precision-recall curves for different variant types representing the precision and recall for distinguishing LOF variants from functional variants. Pangolin achieves a remarkable AUPRC for variants in extended splice regions (note that this excludes canonical splice variants). See Additional file 1: Fig. S8 for variants from additional annotation bins. **f** Predicted splicing effects of mutations in or flanking 4 *BRCA1* exons from Findlay et al. [12]. Mutations identified to be LOF or to have intermediate phenotypes, as well as missense, nonsense, and canonical splice site mutations are annotated. See Additional file 1: Fig. S9 for all 13 exons with predictions. **g** Precision-recall curves representing the precision and recall for distinguishing variants annotated as pathogenic from variants annotated as benign in ClinVar. The blue (resp. orange) line represents the PRC for variants excluding (resp. including) variants in annotated splice sites. Missense and nonsense variants are excluded

As a third use case, we used Pangolin to study the genetic basis of inter-species variation in splice site usage—specifically, variation between human and chimp (note that Pangolin was not trained on chimp data). To this end, we first identified splice sites that are differentially used between human and chimp in brain tissue, focusing on splice sites with a large (≥0.5) difference in usage (“Methods” section, Additional file 2: Table S2). We then asked Pangolin to predict these differences in usage, and found that a cutoff of 0.14 for the predicted differences resulted in a false sign rate of about 5% (Additional file 1: Supplementary Note 7, Fig. S6). Using this cutoff, we were able to identify 35% (550 out of 1560) of the splice sites estimated to have differences in usage greater than 0.5, indicating that sequence divergence proximal (<5 kb) to the splice site contributes to a large fraction of human-chimp differences in RNA splicing. The remaining 65% of the splice sites with large differences in usage may be explained by the effects of mutations more than 5 kb from the splice site or changes in the *trans-*cellular environment, or may represent false negatives that were not detected using Pangolin. Limiting further analysis to substitutions, we found that 47% (97 out of 206) of predicted differences can be explained by a single variant (“Methods” section). A large fraction of these variants create or disrupt a canonical splice site (Fig. 2b,c), as expected, but we also predict that many impact nearby sequences (Fig. 2c). Thus, Pangolin can be used to pinpoint DNA variants responsible for the evolution of splice site usage.

As a last use case, we deployed Pangolin to predict the functional effects of mutations in known disease genes. We reasoned that mutations that are predicted to alter gene splicing are likely to impair function by decreasing functional isoform expression. Thus, accurate identification of mutations that impact splicing may aid in the functional interpretation of variants in disease genes, many of which would otherwise be of uncertain significance. To test this possibility, we first focused our analysis on *BRCA1* single-nucleotide variants whose functional impacts had previously been determined using saturation genome editing (3893 variants in and around 13 exons of *BRCA1*) [12]. We compared the effects of these variants on splicing as predicted using Pangolin to the functional impact of the variants as measured [12]. Strikingly, we found that variants determined to be loss-of-function (LOF) variants were highly enriched among variants predicted to impact RNA splicing (*χ*^2^*p*=6.3×10^−119^, Pangolin cutoff of 0.2), suggesting that impact on splicing is indeed informative for understanding functional impact. To identify the types of variants that drive this enrichment, we classified variants into four categories: non-synonymous, synonymous, splice region (8 intronic bases and 3 exonic bases flanking exon-intron boundaries, excluding non-synonymous and variants in annotated splice sites), or intronic. We found that LOF variants were enriched among variants predicted to affect RNA splicing for all categories, including non-synonymous variants (*χ*^2^ *p*= 4.4×10^−26^, 2.5 × enrichment, Additional file 1: Fig. S7), but particularly for variants in splice regions (*χ*^2^ *p*=1.5×10^−25^,3.1× enrichment, Fig. 2d). Our findings indicate that although LOF variants in splice regions are the most likely to impact splicing, 5–10% of LOF missense variants may impact function through splicing effects rather than by altering protein sequence.

We next sought to directly evaluate Pangolin’s ability to predict LOF variants. This is a difficult task because only a small fraction (823 out of 3893 tested *BRCA1* SNVs) of all possible variants are LOF, and this imbalance generally results in low precision for prediction tasks. Furthermore, many variants, including missense and nonsense variants, are expected to be LOF without affecting RNA splicing. Indeed, using Pangolin’s predicted splicing effects to distinguish LOF variants from functional variants results in a very low AUPRC for missense variants (AUPRC = 0.31). However, when excluding missense and nonsense variants, Pangolin achieves an AUPRC of 0.95 on the remaining 1591 variants, and an AUPRC of 0.90 for the 861 variants in the extended splice region (±15 bp from an exon-intron boundary excluding canonical splice variants, Fig. 2e, “Methods” section). Indeed, mutations predicted to have large impacts on RNA splicing of *BRCA1* appear to correlate particularly well with LOF status throughout the four shown exons (Fig. 2f).

To generalize these findings, we applied Pangolin to variants in extended splice regions from the ClinVar database [20], and found that Pangolin had a similar ability to distinguish the 842 SNVs labeled as pathogenic from the 11,256 SNVs labeled as benign (AUPRC = 0.90, Fig. 2g, “Methods” section), outperforming SpliceAI (AUPRC = 0.87, Additional file 1: Fig. S10). As expected, Pangolin’s performance improved when classifying splice region variants together with variants in annotated splice sites (AUPRC = 0.99, Fig. 2g). Lastly, using Pangolin on 21,363 ClinVar SNVs labeled to be of unknown significance (VUS) in splice regions or annotated splice sites revealed that 5766 VUS are likely to impact splicing and are thus likely pathogenic (Pangolin cutoff of 0.2, Additional file 1: Fig. S11 for *CHEK2* as an example). These results indicate that Pangolin can be used to identify non-missense and non-nonsense pathogenic variants with remarkable accuracy.

In conclusion, Pangolin outperforms contemporary methods for predicting RNA splicing from nearby DNA sequences, can be used for a variety of applications including pathogenic variant prediction, and is available freely online on GitHub (https://github.com/tkzeng/Pangolin).

## Methods

### Deep neural network architecture

Pangolin’s models are dilated convolutional neural networks with an architecture allowing features to be extracted from up to 5000 bases upstream and downstream each target position in the genome. Each model takes as input a one-hot encoded sequence of *N* bases, where *N*≥10,001, and predicts—for the middle *N*−10,000 bases—the probability that these sites are splice sites (probability output model) and the usage of each site (usage output model) in heart, liver, brain, and testis (see “Generating training and test sets” section for details on the format of the input/output, and see Additional file 1: Supplementary Note 1 for characterization of the two model types). In particular, *N* is 10,001 when making predictions for individual sites.

More specifically, the neural networks consist of 16 stacked residual blocks—which are composed of batch normalization, ReLU activation, and convolutional layers—as well as skip connections, which add the model outputs before residual blocks 1, 5, 9, and 13 to the input of the penultimate layer. The convolutional layers of each residual block are dilated—meaning the convolution filter sees every *k*th base, *k*>1, rather than every single base—allowing the receptive field width of the model to increase exponentially with the number of layers. Besides the residual blocks, the networks only contains three other layers—the first and penultimate layers, which are convolutional layers that transform their inputs into the proper dimensions for later layers; and the final activation layer that applies either a softmax or sigmoid activation to produce Pangolin’s probability or usage predictions respectively. In comparison to SpliceAI’s architecture, the last two layers are the primary points of difference—SpliceAI does not make probability/usage predictions for multiple tissues, but rather makes a prediction for the probability that a site is a splice donor, splice acceptor, or not a splice site which is invariable across tissues.

### Generating training and test sets

To identify splice sites and quantify their usages, we processed RNA-seq data from four tissues—heart, liver, brain, and testis—across four species—human, rhesus macaque, mouse, and rat [5]. For heart, liver, and brain, we analyzed 8 samples for each species, while for testis, we analyzed 8 samples for human and 4 for rhesus macaque, mouse, and rat. Samples were chosen from developmental periods subsequent to all periods of large transcriptional changes (Extended Data Fig. 5 from Cardoso-Moreira et al. [5]). RNA-seq reads were mapped to their respective genomes with annotations using STAR 2.7.5 [11] using its multi-sample 2-pass mode (genomes and annotations used: GRCh38 with GENCODE release 34 comprehensive annotations for human; Mmul_10 with ENSEMBL release 100 for rhesus macaque; GRCm38 with GENCODE release M25 for mouse; and Rnor_6.0 with ENSEMBL release 101 for rat). We then assigned multimapped reads to a single location using the multi-mapper resolution (MMR) tool [16].

To create training and test datasets for Pangolin for each tissue, we labeled every position within a gene body as spliced or not spliced and quantified the usage of each splice site. While the first label is binary, the second label—splice site usage—is a continuous value between 0 and 1 representing the proportion of a gene’s transcripts that use a given splice site. Specifically, we labeled all sites within gene bodies supported by 1 split read in at least 2 samples each as spliced, and we labeled all other sites as unspliced. Then, to estimate a splice site’s usage level, we used SpliSER 1.3 [10] to calculate a per-tissue Splice-Site Strength Estimate (SSE). SpliSER considers four types of reads to estimate usage for a target site: *α* and *β*_1_ reads, which are split and non-split reads respectively that map to or across the target site; and *β*_2−SIMPLE_ and *β*_2−CRYPTIC_ reads, which are split reads that provide direct and indirect evidence against usage respectively [10]. Then, SSE is calculated as:

$$\alpha \left/\left(\alpha +\beta_{1}+\beta_{2-\text{SIMPLE}}+\frac{1}{\alpha }\sum_{p\in \{\text{partners}\ \text{of}\ \text{the}\ \text{target}\ \text{site}\}}\alpha_{p}\beta_{2-\text{CRYPTIC},p}\right)\right. $$

We used the SSE metric to estimate the usage of all sites for which *α*+*β*_1_+*β*_2−SIMPLE_≥5 in at least 2 samples. Some sites that we labeled as spliced (1 split read in at least 2 samples) did not meet these criteria and were excluded from both training and testing sets. All sites labeled as not spliced were assigned 0 usage.

For the test set, we set aside genes from human chromosomes 1, 3, 5, 7, and 9, and for the training set, we used all genes from the remaining human chromosomes 2, 4, 6, 8, 10-22, X, and Y that do not show orthology or paralogy to test set genes. We also excluded genes in rhesus macaque, mouse, and rat that show orthology to human test set genes from our training data set. More specifically, we used annotations from Ensembl BioMart (accessed 11/14/2020) to exclude all genes with either low or high “orthology confidence” to a test set gene from the training set.

Next, we prepared the training set as follows. For the model inputs, we extracted the sequence between the annotated 5^′^ most transcription start and 3^′^ most transcription end sites for each gene; padded them with Ns (representing unknown bases) so that each site is surrounded by at least 5000 bases on either side; and split the resulting sequence into overlapping blocks of 15,000 base pairs such that the first block contained positions 0 to 15,000, the second positions 5000 to 20,000, and the *i*th block positions 5000 (*i*−1)− 5000 to 5000 (*i*−1) + 5000. We chose such a block size because it allows many predictions to be made for a single input block (specifically, predictions for the middle 5000 positions), greatly reducing the training time required in comparison to predicting one base at a time, which would require 5000 blocks of 10,001 bases each. We then one-hot encoded each input sequence, representing A, C, G, T/U, and—for unknown bases—N by [1,0,0,0],[0,1,0,0],[0,0,1,0],[0,0,0,1],and [0,0,0,0] respectively.

For the output labels, we assigned each target site a vector of length 12, with positions 0–3, 4–6, 7–9, and 10–12 corresponding to labels for heart, liver, brain, and testis respectively. For each tissue, the first two positions represent whether or not a site is spliced—unspliced sites were labeled as [1,0], spliced sites as [0,1], and padding or unknown sites as [0,0]. The third position is a number between 0 and 1 representing the estimated usage level of the site. For each target site, Pangolin outputs an identically-sized vector of numbers, with each position representing the predicted values.

### Training Pangolin

During training, we randomly held out 10% of the 15,000 base-pair blocks to determine an early-stopping point, which was the epoch when the average training loss stopped decreasing. We first trained the network using the AdamW optimizer and a warm-restarts learning-rate schedule with cycle lengths of 2 and 4 epochs (total training time of 6 epochs) [23]. With this schedule, we initialized the learning rate to 5×10^−4^ at the start of each cycle and decayed it to 0 using a cosine annealing by the end of each cycle. For each input, we computed losses for the model’s probability predictions with a categorical cross-entropy loss function and losses for the model’s usage predictions with a binary cross-entropy loss function; then summed over the losses across all tissues to calculate a total loss. Total losses for the inputs were used to update the model’s weights through backpropagation.

We further trained the model on each tissue and label type (spliced/unspliced and splice site usage) separately so that the loss for each input was computed using only one tissue and label type at a time (we trained each tissue and label type combination for 4 epochs, initializing the learning rate to 5×10^−4^ and decaying it to 0 using a cosine annealing). In addition, we ran the training procedure 5 times, resulting in 5 models per tissue and label type combination (heart-spliced, heart-usage, liver-spliced, liver-usage, etc. for 40 total models). For all predictions of splicing probabilities or splice site usage for a tissue, unless otherwise specified, we took the mean prediction across the 5 models.

Finally, we trained a version of Pangolin by fine-tuning on human data after removing, for each tissue, sequences containing no splice sites; and with the use of label smoothing, a regularization technique wherein unspliced and spliced sites are labeled using the vectors [0.95, 0.05] and [0.05, 0.95] respectively rather than the one-hot encodings [1, 0] and [0, 1] (4 epochs; learning rate was initialized to 5×10^−5^ and decayed to 0 with a cosine annealing). We repeated this training process 3 times to obtain 3 models per tissue. We found that this fine-tuned version of Pangolin generally performed better at predicting splice variants, and use it for the analyses in Figs. 1c, d and 2a, d–g.

### Evaluation on held-out test set

For each tissue, we evaluated Pangolin’s ability to predict splice sites in genes from human chromosomes 1, 3, 5, 7, and 9, excluding genes with low expression levels (mean transcripts per million (TPM) across samples <2.5 as determined using RSEM 1.3.3 [21]). For this evaluation, we used Pangolin’s predictions of tissue-specific splice site probabilities. We first computed, for each tissue, the average top-1 and top-0.5 accuracy over all genes from these chromosomes. The top-1 accuracy is defined as the fraction of sites within the top *N* predicted splice sites that are labeled as splice sites, where *N* is the number of labeled splice sites in the test dataset (i.e., the fraction of the top *N* predicted splice sites that are correct). Similarly, the top-0.5 accuracy is defined as the same fraction but for the top ⌊*N*/2⌋ predicted splice sites. We also computed the area under the precision-recall curve (AUPRC) for each tissue. For SpliceAI (version 1.3.1), we computed the probability that a site is spliced as the maximum of SpliceAI’s 5^′^ and 3^′^ scores. Similarly, for MMSplice (version 2.2.0), we scored each site as the maximum of MMSplice’s 5^′^ and 3^′^ scores following a logit transformation, where the input for each 5^′^ site was the sequence 13 bp into the intron and 5 bp into the exon, and where the input for each 3^′^ site the sequence 50 bp into the intron and 3 bp into the exon. For HAL, we scored each site used the function score_seq_pos (from Cell2015_N8_HAL_Genome_Predictions.ipynb in the GitHub repository https://github.com/Alex-Rosenberg/cell-2015) to obtain 5’ splice site scores, using the sequence 80 bp into the intron and 80 bp into the exon as input. Since HAL does not score 3^′^ splice sites, we excluded 3^′^ splice sites when evaluating HAL’s predictions. Finally, for MaxEntScan, we scored each site as the maximum of MaxEntScan’s 5^′^ and 3^′^ splice scores following the transformation 2^*s*^/(2^*s*^+1) for each score *s*, where the input for the 5^′^ model was the sequence 6 bp into the intron and 3 bp into the exon, and where the input for the 3^′^ model was the sequence 20 bp into the intron and 3 bp into the exon. When running MMSplice, HAL, and MaxEntScan, we excluded inputs that contained “N” bases (unknown bases or padding).

### Definition of maximum difference in probability scores

For some applications of Pangolin, we calculated the splice score of a variant as the maximum difference in probability scores across tissues between the reference and mutated sequence. Here, we define this difference. Let *P*_ref,tissue_ be the predicted probability that a splice site in the reference sequence context is spliced in a tissue, and *P*_alt,tissue_ be this probability for a splice site in the mutated sequence context. Let *Δ*scores be the vector [*P*_alt,heart_−*P*_ref,heart_,*P*_alt,liver_−*P*_ref,liver_,*P*_alt,brain_−*P*_ref,brain_,*P*_alt,testis_−*P*_ref,testis_]. Then, we define maximum difference in probability scores as the element in *Δ*scores corresponding to max|*Δ*scores|, i.e. *Δ*scores_argmax|*Δ*scores|_.

### MFASS and MaPSy evaluation

Cheung et al. [8] used a Sort-seq assay (MFASS) to quantify the effects of 27,733 exonic and intronic variants from the Exome Aggregation Consortium (ExAC) on exon recognition. More specifically, the effects of these variants on splicing were assayed using minigene reporters each containing an exon and its surrounding intronic sequences. Variants with a *Δ*inclusion index of ≤− 0.5 were classified as splice-disrupting variants (SDVs), where *Δ*inclusion index is defined as the difference in percent-spliced-in, the ratio of transcripts containing an exon, between the alternative and reference alleles. To predict the effect of a variant on exon splicing using Pangolin, we took the mean over Pangolin’s scores for the 5^′^ and 3^′^ splice sites of each exon, where each site was scored using the maximum difference in probability scores across tissues between the alternative and reference sequences. Specifically, we used sequences ±5000 bp of the 5^′^ and 3^′^ sites—obtained from the GRCh37 human reference assembly—as the reference sequence inputs to the model, and we used their mutated versions as the alternative sequence inputs. For SpliceAI, we scored each variant as the mean of SpliceAI’s scores for the exon’s 5^′^ and 3^′^ sites, where each site was scored as the difference in score between the alternative and reference alleles. As before, we used the maximum of SpliceAI’s 5^′^ and 3^′^ scores as the score for each site. Variant scores for MMSplice and HAL were previously computed [7]. For Pangolin and SpliceAI, we then computed precision and recall using the precision_recall_curve function from the Python package scikit-learn, and AUPRC using the auc function. For MMSplice, we computed precision and recall using scripts from the MMSplice paper [7] (https://github.com/gagneurlab/MMSplice_paper), and for HAL, we used the precision and recall statistics provided in the MFASS paper [8] (https://github.com/KosuriLab/MFASS).

We further compared the performance of Pangolin, SpliceAI, and MMSplice on the MaPSy dataset [28], obtained from the MMSplice GitHub repository (https://github.com/gagneurlab/MMSplice_paper). Soemedi et al. [28] used a splicing reporter system (MaPSy) to test the effects of 4964 variants from the Human Gene Mutation Database (HGMD) on splicing efficiency, i.e., the proportion of spliced RNAs in the set of total RNAs. They tested the effects of variants both in vitro (cell nuclear extract) and in vivo (HEK293T cells). The effect of a variant on splicing efficiency is calculated as $\log _{2}\left (\frac {m_{o}/m_{i}}{w_{o}/w_{i}}\right)$, where *m*_*o*_ and *w*_*o*_ are the mutant and wild-type spliced-RNA read counts, respectively, and *m*_*i*_ and *w*_*i*_ are the mutant and wild-type unspliced-RNA read counts respectively. For Pangolin and SpliceAI, we scored each variant as the mean of the score for the 5^′^ splice site and the score for the 3’ splice site, following the procedure described above for scoring MFASS variants. We excluded sites for which the measured effect of a variant was undefined (for example, if $\frac {m_{o}/m_{i}}{w_{o}/w_{i}}=0$). We then calculated the Pearson correlations between Pangolin’s and SpliceAI’s predicted scores and the measured effects from MaPSy. For MMSplice, we report the Pearson correlations provided in the MMSplice paper, which were calculated for a smaller test set of variants as MMSplice was trained to predict splicing efficiency using a subset of the variants.

### *FAS* exon 6 evaluation

Julien et al. [15] quantified the effects of all possible single mutations (189 total) in *FAS* exon 6 using a minigene reporter covering *FAS* exons 5–7. In a subsequent study, Baeza-Centurion et al. [3] quantified the effects of several single, double, and higher-order combinations of 12 single mutations (3072 total) in *FAS* exon 6 using the same minigene reporter assay. We used the first dataset to evaluate Pangolin’s performance on single mutations; and used sequences with >1 mutation from the second dataset (3059 out of 3072) to evaluate Pangolin’s performance on multiple mutations. For the first dataset, we converted enrichment scores to PSI estimates by fitting an exponential calibration curve using 24 mutants with experimentally determined inclusion levels. For the second dataset, PSI estimates for each variant were provided in Baeza-Centurion et al. [3]. We scored each variant by computing max(*P*_heart_,*P*_liver_,*P*_brain_,*P*_testis_) for the 5^′^ and 3^′^ splice sites, where *P*_tissue_ is the predicted probability that a site is spliced in the specified tissue. We then used the mean of the scores for the 5^′^ and 3^′^ splice sites to predict exon inclusion levels for each variant. As inputs to Pangolin, we extracted sequences from the GRCh38 reference assembly. To understand the effects of epistatic interactions, Baeza-Centurion et al. [3] developed a linear model with 12 parameters, one for each single base-pair mutation, to predict the PSIs of all exons in the library. For Additional file 1: Fig. S3, we used this model to predict the PSIs for all exons with >1 mutation, and calculated the Spearman’s *r* correlation coefficient between the predicted and observed PSIs.

### Tissue-specific splicing

To evaluate Pangolin’s ability to predict tissue-specific splicing differences, we considered a subset of splice sites in the test genes with higher confidence usage estimates: sites with at least 10 *α*+*β*_1_+*β*_2_ reads per sample (see “Generating training and test sets” section for definitions of the read types) for at least three samples and with standard deviations of <0.1 for the usage estimates. We also required that sites be expressed in all tissues (mean TPM across samples for each tissue ≥2.5) and that for at least one tissue, the splice site usage differs from the mean splice site usage across tissues by >0.2. For each tissue, we computed—for each splice site meeting the above criteria—the observed difference in usage from the mean usage across all tissues. Similarly, we computed each predicted difference as the difference between a splice site’s predicted usage in a tissue and the mean predicted usage across tissues. Then, we computed the Spearman’s *r* correlation coefficient between these predicted and measured differences.

### In silico mutagenesis

We performed in silico mutagenesis by predicting the splicing effects of all possible single base mutations for positions 8 bp into the intron and 4 bp into the exon for 5^′^ splice sites, and for positions 15 bp into the intron and 3 bp into the exon for 3^′^ splice sites. For each splice site, we predicted the effect of each mutation on splice site usage by computing the mean predicted difference across tissues between the reference and mutated sequences. We performed this analysis for the splice sites of protein-coding genes in human chromosomes 7 and 8, limiting our analysis to the most representative transcript per gene as determined by the presence of an Ensembl_canonical tag in the annotation file. Furthermore, we excluded the start of the first exon and end of the last exon of each transcript.

### Splicing QTLs evaluation

To evaluate Pangolin’s ability to predict the effects of common variants in their extant biological contexts, we used Pangolin to distinguish SNPs that are putatively causal for splicing differences—as determined from a splicing QTL (sQTL) analysis—from other nearby SNPs tested in our sQTL analysis. We used a previously analyzed set of sQTLs generated using RNA-seq data from whole blood samples from 922 genotyped individuals in the Depression Genes and Networks (DGN) cohort [24]. For each sQTL, we defined the putatively causal SNP as the SNP with the most significant association (lowest *p* value) with the splicing phenotype out of all tested SNPs. Next, we considered sQTLs whose causal SNPs were within 1000 bp of the intron’s 5^′^ or 3^′^ sites, and analyzed the 500 sQTLs that had the most significant causal SNPs. For each of these sQTLs, we used both Pangolin and SpliceAI to predict the splicing effects of the causal SNP as well as all other SNPs within 1000 bp of the 5^′^ and 3^′^ sites. Specifically, we predicted the effect of each SNP on the nearest splice site by taking the absolute value of the predicted change in splice score. If both the 5^′^ and 3^′^ splice site were within 1000 bp of the SNP, we took the mean over the predictions for both sites; otherwise, we used the prediction for the nearest splice site. For Pangolin, we used the absolute value of the maximum difference in probability score (defined earlier in the “Methods” section) as the prediction for each site.

Next, for Pangolin and SpliceAI, we generated empirical cumulative distribution function (eCDF) plots for the ratio (predicted *p* value)/(putative *p* value), where predicted *p* value for a given QTL is the *p* value of the SNP with the largest predicted effect on splicing as determined by Pangolin or SpliceAI, and putative *p* value is the *p* value of the putatively causal SNP. As baselines (100 in total), we repeatedly selected a random SNP for each QTL and generated eCDF profile for the ratio (random *p* value)/(putative *p* value), where random *p* value is the *p* value of the randomly-chosen SNP.

### Splice site evolution

To predict variants responsible for differences in splice site usage between species, we analyzed RNA-seq data from human, chimpanzee, and rhesus macaque prefrontal cortex [17]. Kanton et al. [17] performed bulk RNA sequencing separately on sliced sections of prefrontal cortex samples. We analyzed two samples per species (one sample per individual), and after combining RNA-seq reads from cortex sections for each sample, mapped reads to their respective genome assemblies with annotations using STAR 2.7.5 in its multi-sample 2-pass mode (assemblies and annotations: GRCh38 with GENCODE release 34 for human; Mmul_10 with ENSEMBL release 100 for rhesus macaque; and Pan_tro_3.0 with ENSEMBL release 101 for chimpanzee). To convert coordinates between genomes, we again used Liftoff [27] to map genomic features from the human genome assembly to the chimpanzee and rhesus macaque genome assemblies, and vice versa. For further analysis, we calculated usage for splice sites with at least 50 *α*+*β*_1_+*β*_2_ reads in each sample and with standard deviations of <0.05 for the usage estimates. We also considered sites that had no reads at all as having 0 usage, and required that sites be in expressed genes (mean TPM across samples ≥2.5). For comparisons of splice site usage between human and chimpanzee, we considered annotated human (resp. chimpanzee) sites that mapped to the chimpanzee (resp. human) genome with alignment coverage ≥0.75 and exon/CDS sequence identity ≥0.75 as determined by Liftoff; were on genes of the same strand; mapped to a single location on the target genome; and were one-to-one orthologs. Next, we considered the differentially used splice sites with |usage in human−usage in rhesus|≥0.5. To score each differentially spliced site using Pangolin and SpliceAI, we computed the maximum difference in predicted splice score between chimp and human using the sequence contexts surrounding the splice site and its lifted-over coordinates as inputs. With these predictions, we then calculated false sign rates (FSR) for a range of predicted score cutoffs (Additional file 1: Supplementary Note 7).

To identify sites where a single mutation is sufficient to explain the difference in splice scores, we first limited analysis to sites predicted to be differentially used (5% FSR, score cutoff = 0.14) for which chimpanzee and human sequences showed at most 10% divergence in regions near the splice site (20 differences within 100 bp upstream and downstream of the splice site). By visualizing the positional distributions of divergent bases, we found that this cutoff kept only sequence differences that are likely to be substitutions (differences were generally isolated to single bases). Next, we kept sites where the predicted difference in usage is explained mostly by these nearby differences rather than by more distal ones (>100 bases from the splice site), and furthermore, where a single nearby mutation is sufficient to explain the difference in splice scores (see Additional file 1: Fig. S12). Examples of such sites are shown in Fig. 2b and c.

### *BRCA1* evaluation and ClinVar prediction

Findlay et al. [12] performed saturation genome editing to test the effects of 3893 SNVs in 13 exons and nearby intronic regions of the *BRCA1* gene (96.5% of all possible SNVs) to determine their functional consequences. Specifically, they performed editing in the HAP1 cell line, where *BRCA1* is essential for cell survival, and calculated variant function scores using depletion of each variant over time from the plasmid library, a metric for cell survival (negative function scores correspond to a decline in *BRCA1* function). For each variant, we used Pangolin to compute the largest decrease in splice score (largest across tissues) at the closest annotated splice site (*BRCA1* transcript BRCA1-203). In addition, if the other splice site for the corresponding exon was within 100 bp, we used the mean predicted decrease across both splice sites as the predicted effect of the variant. We used the GRCh37 reference assembly to extract input sequences for Pangolin. To classify variants as missense, nonsense, intronic, synonymous, splice region, or canonical splice variants, we used the labels provided by Findlay et al. [12]. In particular, variants in splice regions are those that are located up to 3 bp into the exon and 8 bp into the intron that do not disrupt canonical splice sites or alter the amino acid sequence. We define variants in extended splice regions similarly, but include variants ±15 bp of the exon-intron boundary. In addition, we classified variants as loss-of-function (LOF), intermediate, or functional using the function score thresholds determined in Findlay et al. [12]. For all analyses, such as computing precision-recall curves and AURPC, we considered only LOF and functional variants.

The ClinVar database contains variants found in patient samples, many of which are classified as Pathogenic, Likely pathogenic, Likely benign, Benign, or Uncertain Significance. We applied Pangolin to ClinVar variants downloaded from https://ftp.ncbi.nlm.nih.gov/pub/clinvar/vcf_GRCh38/clinvar.vcf.gz on 05/04/2021. Specifically, for all variants passing certain criteria (listed below), we computed the maximum decrease in splice score at an annotated splice site within 50 bases of the variant, using the GRCh38 genome assembly and GENCODE Release 38 gene annotations filtered for the most representative (Ensembl_canonical tagged) transcripts. These criteria were: the variant is a substitution or simple insertion/deletion (insertion/deletion where either the REF or ALT field is a single base); is contained in a gene body; is not within 5000 bases of the chromosome ends; and is not a deletion larger than 100 bp. We also ran SpliceAI on the same variants, similarly by computing the maximum decrease in splice score at an annotated splice site within 50 bases of the variant (same genome and annotations). For further analyses, we considered variants in protein-coding genes that were either classified by ClinVar as Benign, Pathogenic, Likely Benign, Likely Pathogenic, or Uncertain significance; and required that each variant be in only one gene, not be a nonsense or missense variant as determined using the molecular consequence (MC) field in the ClinVar VCF (variants with no such field were excluded), and be within 15 bp of an annotated splice site (excluding the start of the first exon and end of the last exon of each transcript). We also only considered variants that could be scored by both Pangolin and SpliceAI. To distinguish between variants in annotated splice sites and all other variants, we looked for the presence of the splice_acceptor_variant and splice_donor_variant tags in the MC field.

## Supplementary Information

**Additional file 1** **Supplementary Note 1:** Binary versus continuous prediction output. **Supplementary Note 2:** Identifying a test set with minimal similarity to the training data. **Supplementary Note 3:** Training Pangolin using quantitative splicing data from multiple species improve prediction. **Supplementary Note 4:** Pangolin versus MTSplice on predicting tissue-type-specific splicing. **Supplementary Note 5:** Identifying motifs involved in tissue-specific splicing. **Supplementary Note 6:** Mutations away from G at the -1 position of the 5’ splice site cause strong decreases in 5’ splice site usage. **Supplementary Note 7:** Predicting causal variants that explain inter-species divergence in splice site usage. **Figure S1:** Precision and recall at different distances from a splice site. Figure S2: Pangolin scores correlate with changes in splicing efficiency. **Figure S3:** Prediction of epistatic effects on RNA splicing as a combination of single SNP effects. **Figure S4:** Prediction of tissue specific splice site usage using Pangolin. **Figure S5:** Motifs characterizing tissue-specific splice sites. **Figure S6:** False sign rates of predicted causal variants underlying inter-species divergence in splice site usage. **Figure S7:** Survival function plots of tested *BRCA1* variants. **Figure S8:** Precision and recall for *BRCA1* predictions and fraction LOF at different cutoffs. **Figure S9:** Predicted effects of variants in *BRCA1*. **Figure S10:** Precision and recall for pathogenic versus benign variant classification using Pangolin versus SpliceAI. **Figure S11:** Predicted effects of variants in *CHEK2*. **Figure S12:** Schematic for identifying single causal variants. **Figure S13:** Comparison of training on binary classification of splice sites versus continuous usage estimates. **Figure S14:** Comparison between Pangolin and SpliceAI for predicting inter-species variation in splice site usage. **Table S3:** Evaluations on subsets of test genes. **Table S4:** Comparison of AUPRC between models trained on multiple species and on human only.

**Additional file 2** **Table S1:** Mapping statistics for RNA-seq datasets from Cardoso-Moreira et al., 2019 used for Pangolin training and testing. **Table S2:** Mapping statistics for RNA-seq datasets from Kanton et al., 2019 used in the chimp-human splice site divergence analysis.

**Additional file 3** Review history.

## Data Availability

RNA-seq reads for primary model training and evaluation are available through ArrayExpress (mouse: https://www.ebi.ac.uk/arrayexpress/experiments/E-MTAB-6798, rat: https://www.ebi.ac.uk/arrayexpress/experiments/E-MTAB-6811, rhesus macaque: https://www.ebi.ac.uk/arrayexpress/experiments/E-MTAB-6813, human: https://www.ebi.ac.uk/arrayexpress/experiments/E-MTAB-6814. RNA-seq reads used in the splice site evolution analysis are available through ArrayExpress (rhesus macaque, chimpanzee, and human: https://www.ebi.ac.uk/arrayexpress/experiments/E-MTAB-8231). MFASS data are available on GitHub at https://github.com/KosuriLab/MFASS. *FAS* exon 6 data are available in Supplementary Data 1 of Julien et al. [15]. Processed DGN data are available through Mu et al. [24]. *BRCA1* data are available in Supplementary Table 1 of Findlay et al. [12]. Pangolin is available under a GPL-3.0 License on GitHub at https://github.com/tkzeng/Pangolin and Zenodo [32].
