# Supplementary material for: Predicting RNA splicing from DNA sequence using Pangolin
Source: Genome Biol. 2022 Apr 21;23:103. doi: 10.1186/s13059-022-02664-4 (PMC9022248; doi:10.1186/s13059-022-02664-4)
Supplement: Supplementary file 1 — Additional file 1 Supplementary Note 1: Binary versus continuous prediction output. Supplementary Note 2: Identifying a test set with minimal similarity to the training data. Supplementary Note 3: Training Pangolin using quantitative splicing data from multiple species improve prediction. Supplementary Note 4: Pangolin versus MTSplice on predicting tissue-type-specific splicing. Supplementary Note 5: Identifying motifs involved in tissue-specific splicing. Supplementary Note 6: Mutations away from G at the -1 position of the 5’ splice site cause strong decreases in 5’ splice site usage. Supplementary Note 7: Predicting causal variants that explain inter-species divergence in splice site usage. Figure S1: Precision and recall at different distances from a splice site. Figure S2: Pangolin scores correlate with changes in splicing efficiency. Figure S3: Prediction of epistatic effects on RNA splicing as a combination of single SNP effects. Figure S4: Prediction of tissue specific splice site usage using Pangolin. Figure S5: Motifs characterizing tissue-specific splice sites. Figure S6: False sign rates of predicted causal variants underlying inter-species divergence in splice site usage. Figure S7: Survival function plots of tested BRCA1 variants. Figure S8: Precision and recall for BRCA1 predictions and fraction LOF at different cutoffs. Figure S9: Predicted effects of variants in BRCA1. Figure S10: Precision and recall for pathogenic versus benign variant classification using Pangolin versus SpliceAI. Figure S11: Predicted effects of variants in CHEK2. Figure S12: Schematic for identifying single causal variants. Figure S13: Comparison of training on binary classification of splice sites versus continuous usage estimates. Figure S14: Comparison between Pangolin and SpliceAI for predicting inter-species variation in splice site usage. Table S3: Evaluations on subsets of test genes. Table S4: Comparison of AUPRC between models trained on multiple species and on human o [file 13059_2022_2664_MOESM1_ESM.pdf]

Supplementary Materials for
Predicting RNA splicing from DNA sequence using Pangolin

Supplementary Notes

Supplementary Note 1: Binary versus continuous prediction output

SpliceAI outputs the probability that a dinucleotide is a splice site. We sought to test whether directly predicting splice site usage can improve prediction of splice site usage as compared to using the probability that a dinucleotide is a splice site as usage. To do this, we first placed sites into bins of usage [0, 0.1], (0.1, 0.5], (0.5, 0.9], and (0.9, 1]. For sites in adjacent bins (for example, sites with usage between (0.1, 0.5] or between (0.5, 0.9]), we used Pangolin's probability and usage outputs to predict which of the two bins the sites belonged to (Fig. S13). Directly predicting splice site usage led to an improvement in AUPRC of 0.018 to 0.050 (median: 0.025). Violin plots show differences in the distributions of the probability and usage predictions for each bin (Fig. S13). Depending on the application, we used either Pangolin's probability or usage predictions (Methods).

Supplementary Note 2: Identifying a test set with minimal similarity to the training data

We constructed Pangolin's training set (Methods) such that all genes with annotated orthologs and paralogs from rat, mouse, and rhesus macaque in the test-set genes were removed. Nevertheless, genes with *some* homology to test-set genes may have been included in the training set (e.g. due to being unannotated, distant paralogs). To evaluate Pangolin on a subset of genes with stricter filtering, we used Liftoff, a genome annotation lift-over tool, to map human genes to the mouse, rat, and rhesus macaque genomes [31]. Liftoff uses the Minimap2 aligner to align gene sequences to a target genome, finding the alignment of exons and coding DNA sequence (CDS) that maximizes sequence identity while preserving gene structure (referred to as alignment coverage). Furthermore, Liftoff is able to identify sequence homologous to a gene. To remove test-set genes with similarity to training-set genes, we ran Liftoff using two sets of cutoffs for sequence identity (parameter -s) and alignment coverage (parameter -a), and removed genes that mapped to mouse, rat, and rhesus macaque genomes with identity/coverage greater than these cutoffs. 3,037 genes (29%) of the genes from our original test set passed a cutoff of 0.4 for both alignment coverage and sequence, while 1,585 genes (15%) passed a cutoff of 0.2.

For Pangolin and SpliceAI, we computed top-1 accuracies and AUPRC on these subsets (Table S3—mid sim and low sim correspond to the 0.4 and 0.2 cutoff respectively). The improvements of Pangolin over SpliceAI were similar for the original, mid sim, and low sim test sets: 5.2%, 4.1%, and 5.0% increases in top-1 accuracy, and 10.6%, 10.2%, and 11.5% increases in AUPRC respectively. These results indicate that Pangolin's improvements do not come from "memorization"

of homologous sequences from the training data.

Supplementary Note 3: Training Pangolin using quantitative splicing data from multiple species improve prediction

To identify the features of Pangolin responsible for its increased performance over SpliceAI, we trained four additional models:

1. A model trained on human data only, with prediction on merged tissues with no usage labels—only binary labels of spliced or unspliced. We merged tissues by taking the union of splice sites across tissues. This model is most similar to that of SpliceAI, which was trained to predict binary labels in a tissue-agnostic manner.

2. A model trained on data from all species, with prediction on merged tissues with no usage labels.

3. A model trained on data from all species, with prediction on merged tissues and with usage labels. We trained this model to predict both the existence and usage of splice sites. We merged per-tissue usage labels by taking the average across tissues for which usage labels were available.

4. A model trained on data from all species, with separate predictions for each tissue and with usage labels. This model is equivalent to the full Pangolin model, except we train it without fine-tuning on each individual tissue to allow for comparisons to models 1-3.

For each model, we trained until the validation loss stopped decreasing, which took 14 epochs for model 1 and 6 epochs for the other models, following the training procedure described in Methods except without the tissue-specific fine-tuning step. We repeated the training procedure twice, and found that there was minimal variance between the replicates (Table S4). Furthermore, as expected, we found that model 1 produces similar results as SpliceAI (average AUPRC of 0.764 for model 1, AUPRC of 0.765 for SpliceAI). Training on data from multiple species improved predictions (AUPRC increase of 2.6%, model 2 over model 1), as did training with usage labels (AUPRC increase of 2.4%, model 3 over model 2) and having separate output heads for each tissue (AURPC increase of 1.8%, model 4 over model 3). These results demonstrate that each of the model additions we made to Pangolin (compared to SpliceAI's models) led to consistent improvements in predictive performance.

Supplementary Note 4: Pangolin versus MTSplice on predicting tissue-type-specific splicing

Cheng et al. [8] developed a deep learning model, MTSplice, for predicting tissue-specific effects of variants on splicing. MTSplice consists of MMSplice [6] and TSplice, a deep learning model that

considers 100 bases into the exon and 300 bases into the neighboring introns to predict tissue-specific percent spliced-in (PSI). Cheng et al. [8] evaluated TSplice by computing—for each tissue—the Spearman's r correlation coefficient between the observed and predicted log odds ratios of tissue specific PSI for test set exons for which PSI deviated from the tissue-averaged PSI by at least 0.2 in at least one tissue and for which the corresponding gene is expressed in at least 10 tissues (out of 51 total tissues). We evaluated Pangolin in an analogous fashion, but measured Spearman correlation for observed and predicted differences in splice site usage rather than differences in PSI (Methods). Due to differences in the phenotypes being predicted; the number of tissues evaluated (4 for Pangolin, 51 for MTSplice); the datasets used for evaluation; and other differences in implementation, Pangolin's correlations are not directly comparable to those of TSplice.

Supplementary Note 5: Identifying motifs involved in tissue-specific splicing

To characterize the sequence features that Pangolin has learned to predict tissue-specific splicing, we set to identify motifs that distinguish between predictions for tissue-specific and non-specific splice sites. First, using DeepLIFT [33], a neural network feature-attribution method, we assigned importance scores to each base in the sequences surrounding tissue-specific splice sites (sequences $\pm$500 bp) as predicted using Pangolin. In particular, we ran DeepLIFT on 663 brain- and 1,738 testis-specific splice sites that Pangolin correctly predicted. We considered a tissue-specific site to be correctly predicted if the measured and predicted differences in usage from the mean usage across tissues were both $>$0.05. Since there were many fewer correctly predicted heart- and liver-specific splice sites (60 and 46 splice sites respectively), we did not conduct analyses for these tissues. To compute importance scores, DeepLIFT compares the target input against a reference or baseline input, which we set as a vector of all zeros (corresponding to a sequence of all N's, or unknown bases). Additionally, for both brain and testis, we ran DeepLIFT on five random sets of non-tissue-specific splice sites that had similar distributions in their usage levels as the corresponding tissue-specific splice sites—these splice sites served as controls in our later enrichment analyses.

Next, we identified clusters of high-importance bases by calling peaks using MACS [34], using an absolute importance score cutoff of 0.001, minimum peak size of 5, and maximum allowed gap size of 10 as input parameters. Importantly, although MACS is generally used for finding peaks in genomics data (e.g. ChIP-seq, or ATAC-seq), visual inspection of the peaks identified using this approach on DeepLIFT scores appeared to be consistent with high-scoring regions that we would annotate by hand. After extending each peak by 5 bases on each side, we conducted a differential enrichment analysis on the sequences underlying the peaks to identify motifs enriched in brain- and testis-specific splice sites relative to the control splice sites. To do this, we used the Multiple Em for Motif Elicitation (MEME) Suite [35]. Specifically, with the MEME tool in its differential enrichment mode, and allowing for any number of motif repetitions to occur in each input sequence, we searched for motif enrichment in tissue-specific sequences against each of the five sets of control

sequences. In addition, for five pairs of control sequence sets, we searched for motif enrichment in control sequences against control sequences.

At a MEME E-value cutoff of 0.01, we found zero significant motifs from comparisons of control against control sequences, but five differentially enriched motifs in brain-specific splice sites and six in testis-specific splice sites. Next, we characterized the top five differentially enriched motifs from each tissue. We first noticed that the motifs for both brain and testis look roughly similar (Fig. S5). To analyze this further, we calculated pairwise Pearson correlations between the motifs for a given tissue (within-group similarity) and correlations between brain and testis motifs (across-group similarity), and found that they were moderate but not substantially different from each other (r = 0.42 for the brain motifs, 0.53 for the testis motifs, and 0.48 between the brain and testis motifs). This indicates that the brain and testis motifs may share common features.

To directly test for differences between motifs for brain- and testis specific splice sites, we used MEME to identify differentially enriched motifs in brain sequences relative to testis sequences and vice versa. We conducted this analysis both for brain- and testis- specific sites, and for sets of control sites (comparing brain control sites against testis control sites and vice versa). Two motifs were significantly enriched—one in testis-specific sites relative to brain-specific sites, and one in a set of testis control sites relative to a set of brain control sites. We conclude that it is unclear whether Pangolin has learned sequence features that distinguish testis-specific sites from brain-specific sites, or broader differences in splicing between the two tissues.

Next, we looked at the positional distributions of the top five differentially enriched motifs from each tissue. Using FIMO, a tool from the MEME Suite, we scanned the motif across 1,000 bases surrounding each tissue-splice site, and plotted histograms of the locations of the significant motif hits (Fig. S5). While significant hits are unevenly distributed across the entire range of positions, they are generally low in number near the splice site. For many pairs of motifs, the distributions are roughly similar, indicating that these motifs capture similar sequence features (Fig. S5).

Finally, we tested more stringent cutoffs for identifying motifs. First, we identified sharper peaks by running MACS using an absolute importance score cutoff of 0.005 and maximum allowed gap size of 2. Second, instead of using MACS, we identified peaks using a sliding-window approach, where we computed the average importance scores over four bp windows, kept windows with average scores $\geq$0.005, and merged overlapping windows—we used the resulting windows as peaks. For both approaches, we searched for differential enrichment of motifs over a tighter motif size range than before (8-15 bp using STREME from the MEME Suite). In comparing testis-specific sites to sets of control sites, we found one motif at an E-value cutoff of 0.05 using each approach (Fig. S5). We found no differentially enriched motifs for the control against control or the brain-specific against control comparisons.

Supplementary Note 6: Mutations away from G at the -1 position of the 5' splice site cause strong decreases in 5' splice site usage

The G is complementary with U1 snRNA at the -1 position. Interestingly, the effect of mutating away from G does not reciprocate mutating to a G. We hypothesize that mutations on well spliced introns in general have more room to decrease their splicing efficiency than increase it. This observation also holds for the -3 position at the 3' splice site.

Supplementary Note 7: Predicting causal variants that explain inter-species divergence in splice site usage

To predict causal sequence differences underlying inter-species divergence in splicing, we used Pangolin to predict the effects of human-chimpanzee sequence differences near splice sites with large differences in usage between human and chimpanzee. We calculated a false sign rate (FSR) for different Pangolin score thresholds, which corresponds to the fraction of sites for which the predicted differences in splice site usage are of opposite directions from those of the observed differences. We found that at a fixed FSR, Pangolin was consistently able to predict the correct directions of effects (and thus the likely causal variants) for more splice sites in comparison to SpliceAI (Fig. S14).

As validation, we sought to estimate the splice site usage of human-chimpanzee divergent sites in rhesus macaque. We reasoned that if the mutation predicted to explain human-chimp divergence in splicing occurred in the human (or chimp) lineage, then splice site usage in rhesus macaque would be closer to that in chimp (or human). To test this, we first obtained 46 differentially used splice sites (5% FSR, cutoff = 0.14) for which chimpanzee, human, and rhesus macaque sequences showed at most 10% divergence in regions near the splice site (20 differences within 100 bp upstream and downstream of the splice site, pairwise comparisons between species). Out of these, we identified 17 sites where a single mutation sufficiently explains the predicted human-chimp difference in usage (Methods); for 16 of these sites, the rhesus macaque sequence at the location of the causal variant matched either the human or chimpanzee sequence. We found that for 14 of these 16 splice sites (88%), the predicted differences in splice site usage between human and chimpanzee were consistent with splice site usage measured in rhesus macaque. We considered a prediction to be consistent if the mutation occurred in the human lineage and the splice site's usage in rhesus is more similar to that in chimp than that in human, or the mutation occurred in the chimp lineage and the splice site's usage in rhesus is more similar to that in human than that in chimp.

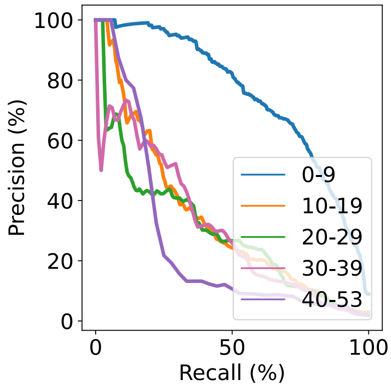

Distance	# variants	% SDV	Pangolin, AUPRC	SpliceAI, AUPRC
0-9	6141	8.8	75	68
10-19	7559	2.9	34	25
20-29	7052	2.2	29	18
30-39	4953	2.0	31	18
40-53	2028	1.8	25	13

Fig. S1. Precision and recall at different distances from a splice site. The precision-recall curves show the precision and recall for predicting splice-disrupting variants in the MFASS dataset when restricted to variants in a given distance bin from a splice site (0-9, 10-19, 20-29, 30-39, and 40-53 bases from a splice site). For each distance bin, the table lists the total number of variants, the percent that are splice-disrupting variants (SDV), and AUPRC for Pangolin and SpliceAI—Pangolin outperforms SpliceAI for all distance bins.

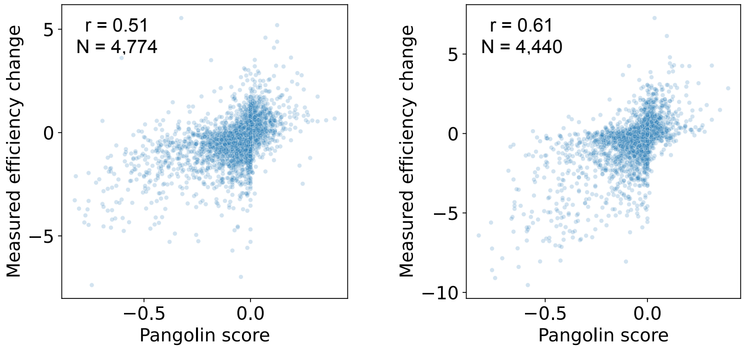

Method	Pearson r, *in vitro*	Pearson r, *in vivo*
Pangolin	0.51	0.61
SpliceAI	0.40	0.50
MMSplice	0.57	0.37

Fig. S2. Pangolin scores correlate with changes in splicing efficiency. Scatter plots showing the correspondence between the predicted effect of a variant (x-axis) and the measured change in splicing efficiency (y-axis) for variants tested *in vitro* (top left) and *in vivo* (top right) using MaPSy. The table lists the Pearson correlations for Pangolin, SpliceAI, and MMSplice for both assays—MMSplice achieves the highest correlation for the *in vitro* assay, while Pangolin achieves the highest correlation for the *in vivo* assay. Note that the correlations for MMSplice may be inflated relative to that of Pangolin and SpliceAI because only SNPs near the splice sites were predictable using MMSplice.

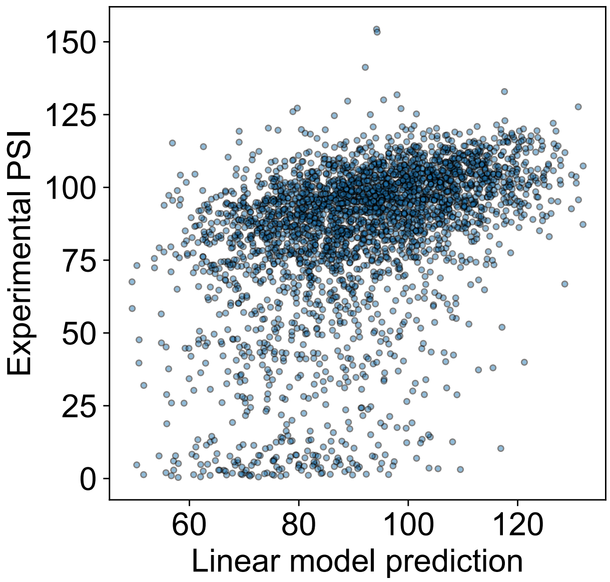

Fig. S3. Prediction of epistatic effects on RNA splicing as a combination of single SNP effects. Scatter plot showing measured (y-axis) versus predicted (x-axis) effects of combinations of genetic variants on RNA splicing. Measured effects of combinations of variants were obtained from Baeza-Centurion et al. [18]. Predictions were made using a linear model of the single variant PSIs (see Methods).

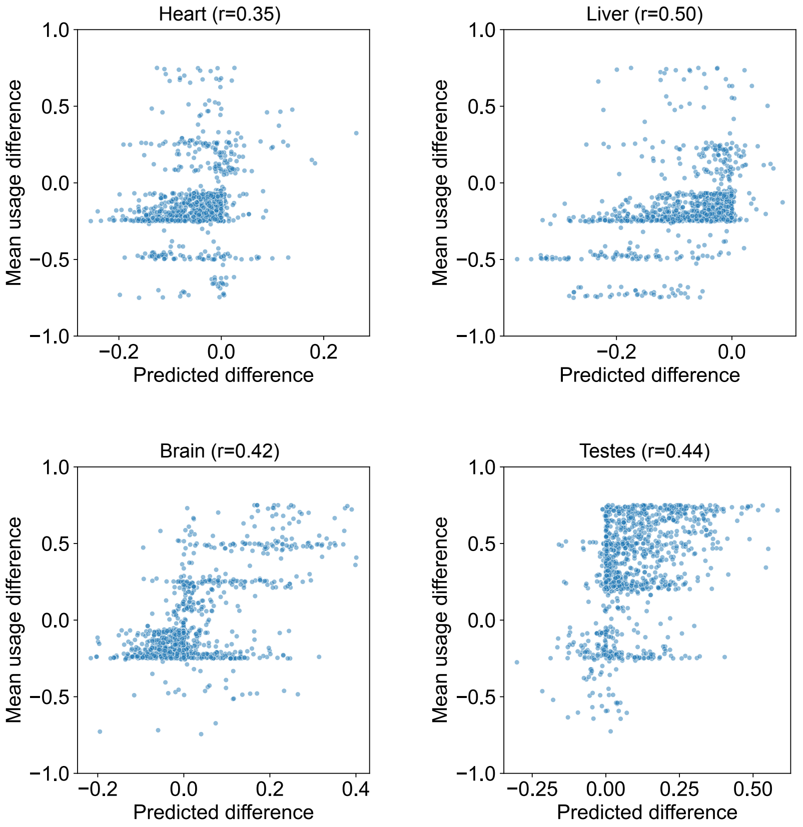

Fig. S4. Prediction of tissue-specific splice site usage using Pangolin. Scatter plots showing the mean empirical difference between splice site usage in the specified tissue and mean usage across all tissues (y-axis) versus the predicted difference as determined using Pangolin (x-axis). The prediction accuracies, although low, outperform or are comparable to those of MTSplice [8]. Our results suggest that predicting tissue-specific splicing remains challenging.

ID	E-value	Motif
testis 1	4.70E-152	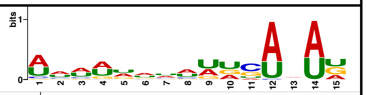
testis 2	1.20E-184	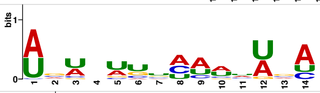
testis 3	6.10E-211	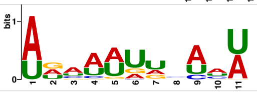
testis 4	2.30E-174	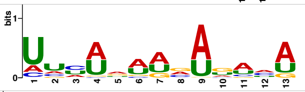
testis 5	6.80E-196	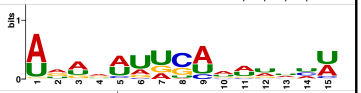
testis MACS strict	4.7E-02	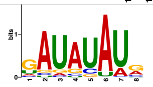
testis sliding window	2.0E-02	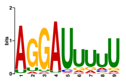

ID	E-value	Motif
brain 1	2.3E-34	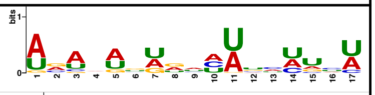
brain 2	8.8E-54	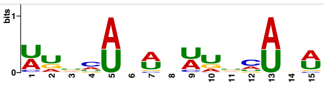
brain 3	9.2E-38	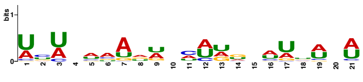
brain 4	6.3E-35	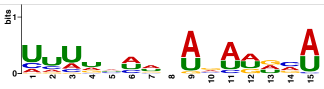
brain 5	5.4E-41	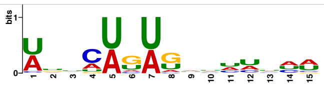

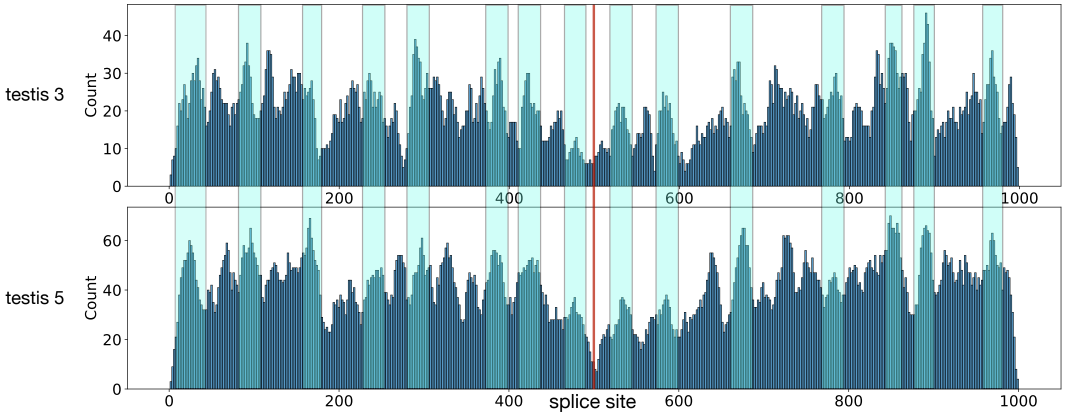

Fig. S5. Motifs characterizing tissue-specific splice sites. Tables display E-values and sequence logos (MEME) for the top motifs discovered for testis- (top left) and brain-specific splice sites (top right). For testis, also shown are the two motifs identified using alternative sets of peak calls (testis MACS strict and testis sliding window). The bottom plot shows the positional distributions of significant hits from scanning the testis 3 and testis 5 motifs against the sequences surrounding testis-specific splice sites. Light blue bars highlight some of the peaks that are found in both distributions.

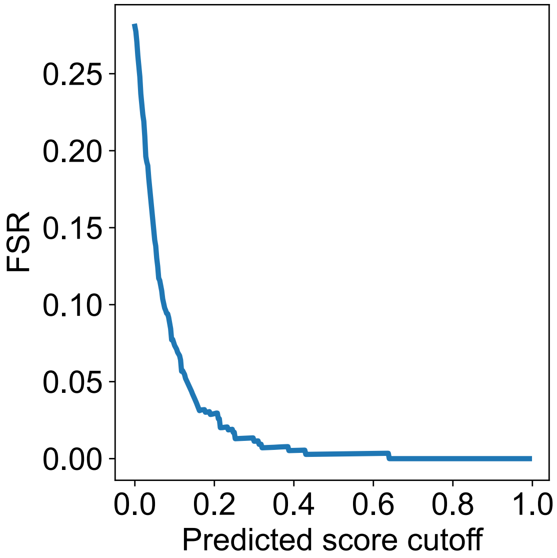

Fig. S6. False sign rates of predicted causal variants underlying inter-species divergence in splice site usage. False sign rates (FSR) at different cutoffs for predicted scores determined using Pangolin, calculated across 1,560 splice sites with large differences in usage (≥ 0.5) between human and chimpanzee. We observed a FSR of about 5% at a cutoff of 0.14.

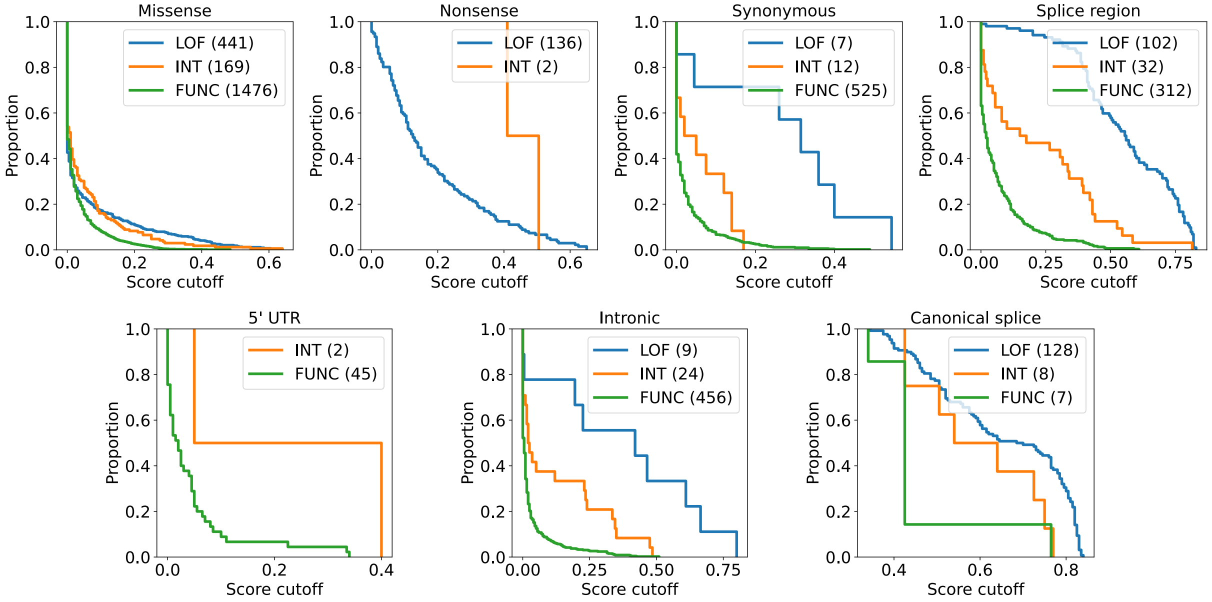

Fig. S7. Survival function plots of tested *BRCA1* variants. Survival function plots of *BRCA1* variants in different annotation bins as a function of predicted splicing effects. The variants are separated by their classification as loss-of-function (LOF, blue), intermediate (INT, orange), or functional (FUNC, green). We observe a huge enrichment of LOF variants among variants with large predicted splicing effects for several annotation classes, with splice regions the most enriched and missense the least enriched. Functional data are from Findlay et al. [22].

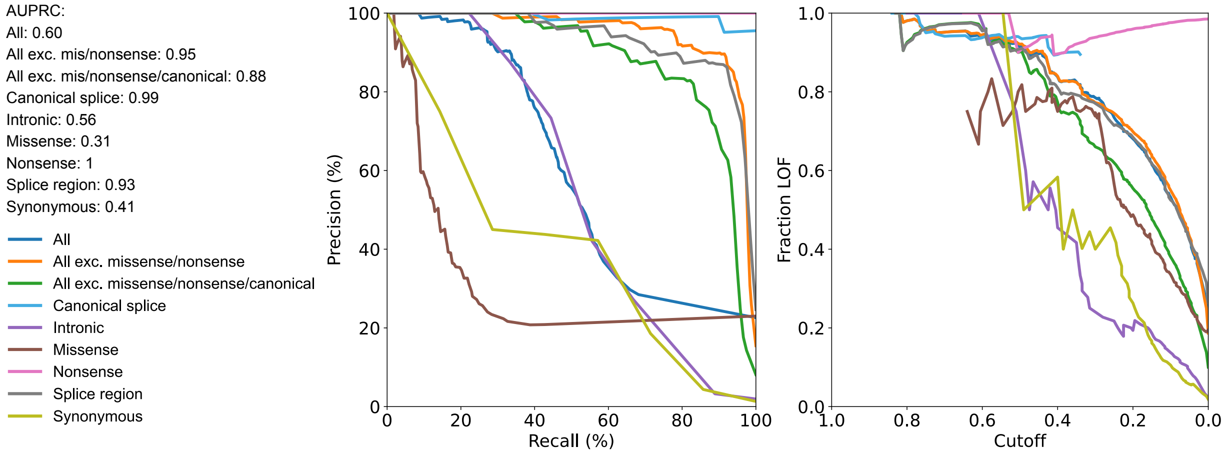

Fig. S8. Precision and recall for *BRCA1* predictions and fraction LOF at different cutoffs. Left panel: Precision-recall curves, one for the variants in each annotation bin, representing the precision and recall for using Pangolin predictions to distinguish loss-of-function (LOF) variants from functional variants. Right Panel: Line plots showing the fraction of variants classified as LOF as a function of Pangolin's predicted effects on splicing. Despite a poor AUPRC for missense variants, Pangolin maintains a roughly 60% and 80% precision for variants with predicted effects on splicing greater than 0.4 and 0.8 respectively.

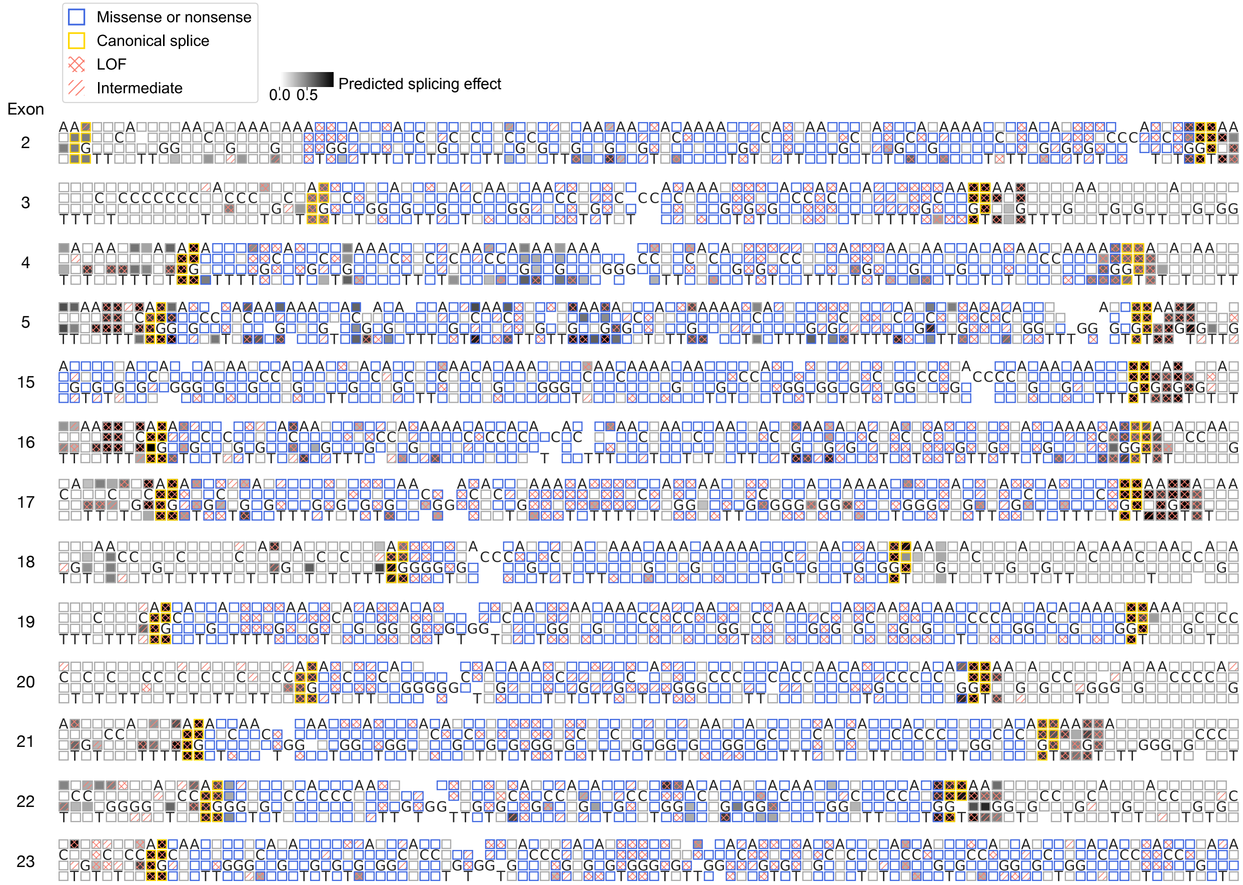

Fig. S9. Predicted effects of variants in *BRCA1*. Predicted splicing effects of *in silico* mutations in or flanking 13 *BRCA1* exons from Findlay et al. [22]. Mutations identified to be LOF or to have intermediate phenotypes, as well as mutations that are missense or nonsense, or affect the canonical splice sites are annotated.

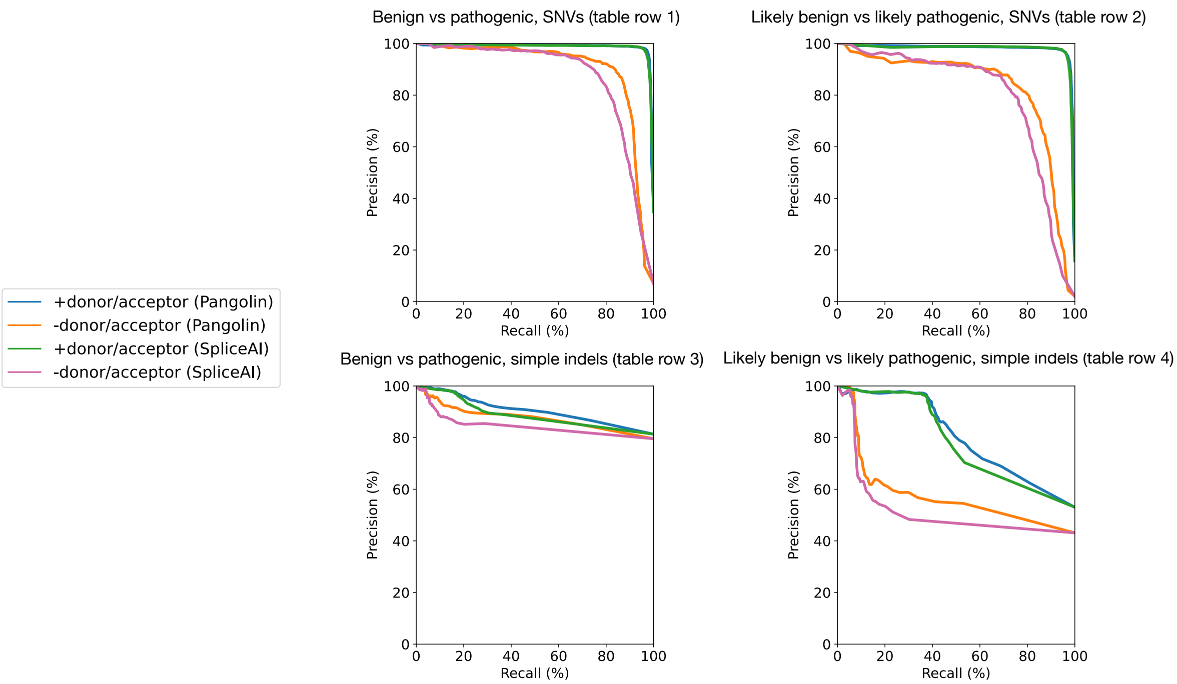

		+donor/acceptor				-donor/acceptor			
		AUPRC, Pangolin	AUPRC, SpliceAI	N (likely) pathogenic	N (likely) benign	AUPRC, Pangolin	AUPRC, SpliceAI	N (likely) pathogenic	N (likely) benign
SNVs	Benign vs pathogenic	0.99	0.99	6,050	11,316	0.90	0.87	842	11,256
	Likely benign vs likely pathogenic	0.98	0.98	5,455	28,961	0.83	0.80	597	28,864
Simple indels	Benign vs pathogenic	0.90	0.89	5,153	1,182	0.88	0.84	4,378	1,125
	Likely benign vs likely pathogenic	0.80	0.78	1,910	1,693	0.57	0.52	1,218	1,608

Fig. S10. Precision and recall for pathogenic versus benign variant classification using Pangolin versus SpliceAI. Top: Precision-recall curves for distinguishing ClinVar variants annotated as pathogenic versus benign (left) or likely pathogenic versus likely benign (right) for SNVs (top) and simple indels (bottom, indels where the reference or alternative allele is only a single base) using Pangolin and SpliceAI, including (+donor/acceptor) or excluding (-donor/acceptor) variants affecting annotated splice sites. Bottom: Table displaying AUPRC for each precision-recall curve, and the number of variants in each of the classes represented in the curves.

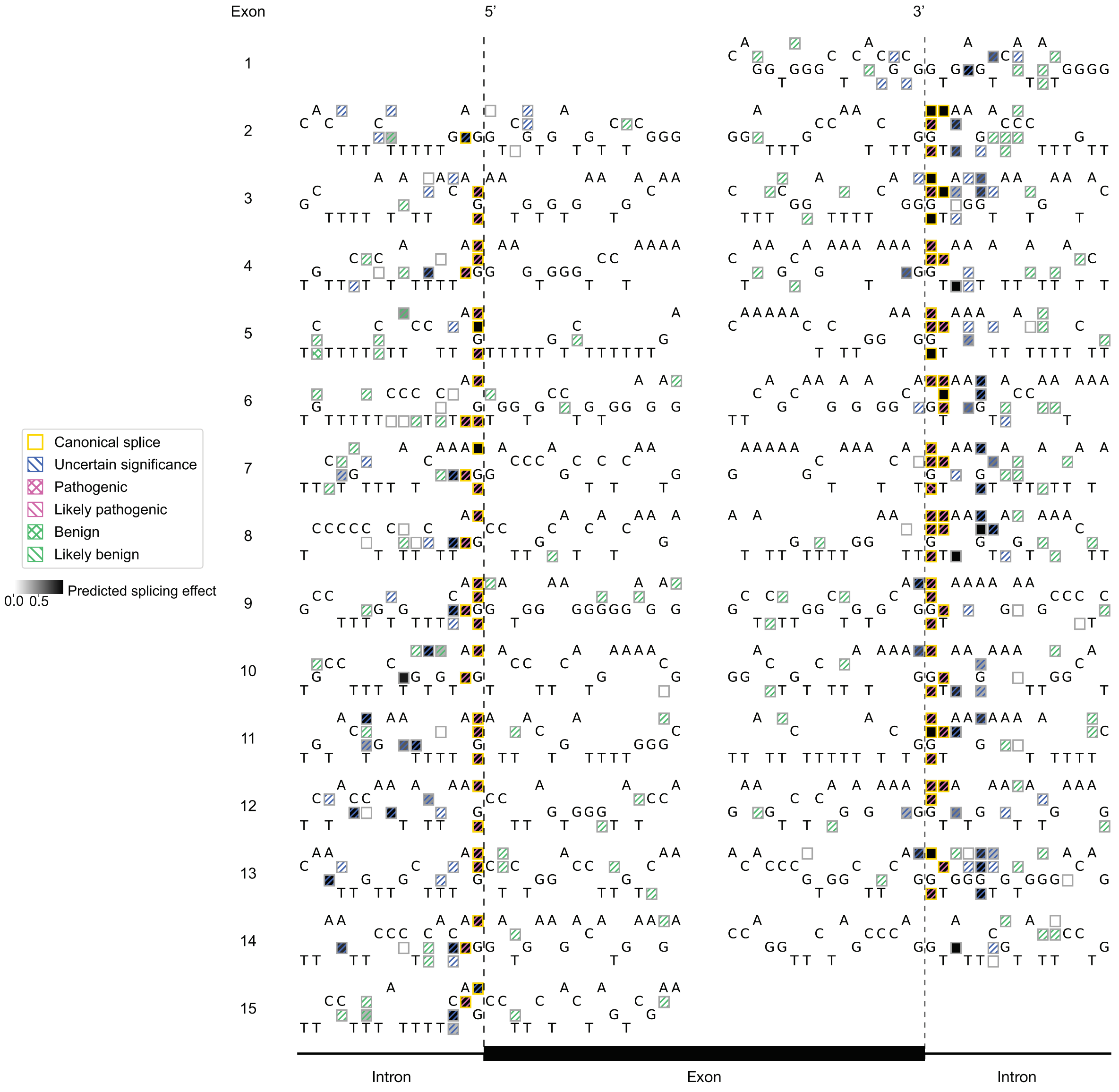

Fig. S11. Predicted effects of variants in *CHEK2*. Many variants of unknown significance, as labeled by ClinVar, are predicted to impact splicing and therefore are likely pathogenic.

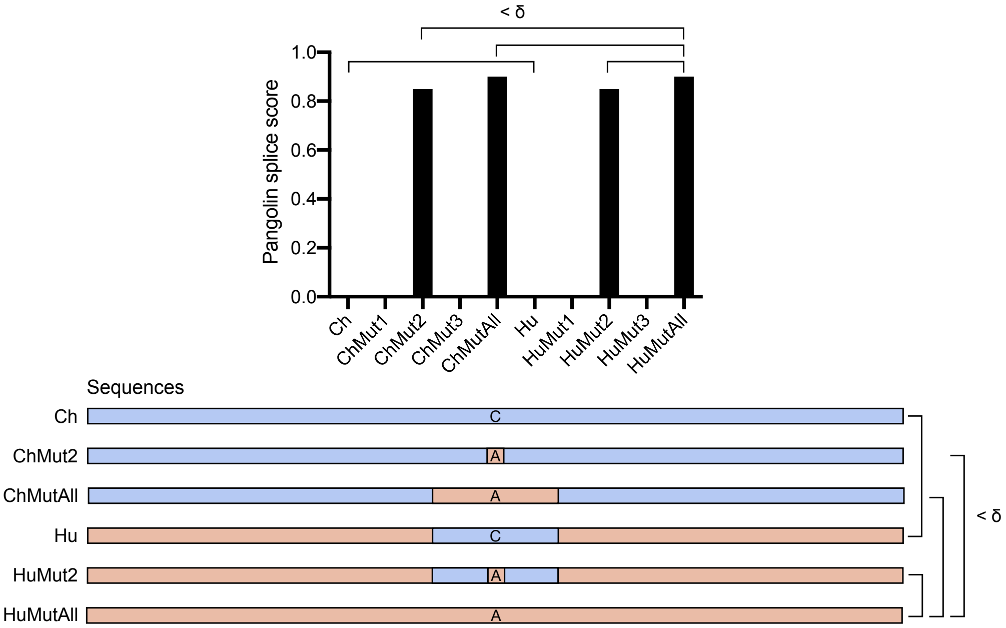

Fig. S12. Schematic for identifying single causal variants. The schematic shows a hypothetical example where three SNVs (Mut1-3) cause in a large increase in the predicted usage of a splice site; specifically, Mut2 is predicted to be the only causal SNV. Ch is the chimp sequence ±5000 bp of the splice site, ChMut2 is Ch with Mut2, and ChMutAll is Ch with Mut1-3. Hu is the human sequence ±5000 bp of the splice site but with the region near the splice site (±100 bp) replaced by the chimp sequence. HuMut2 and HuMutAll are Hu with Mut2 and Mut1-3 respectively. We say that Mut2 is the single causal mutation for this splice site if $|\text{score}_{\text{Ch}} - \text{score}_{\text{Hu}}| < \delta$, $|\text{score}_{\text{ChMutAll}} - \text{score}_{\text{HuMutAll}}| < \delta$, $|\text{score}_{\text{ChMutX}} - \text{score}_{\text{HuMutAll}}| < \delta$, and $|\text{score}_{\text{HuMutX}} - \text{score}_{\text{HuMutAll}}| < \delta$ for X = 2 but not for X = 1 or X = 3, where we define δ as $\min(0.1, |\text{score}_{\text{ChMutAll}} - \text{score}_{\text{HuMutAll}}|/5)$.

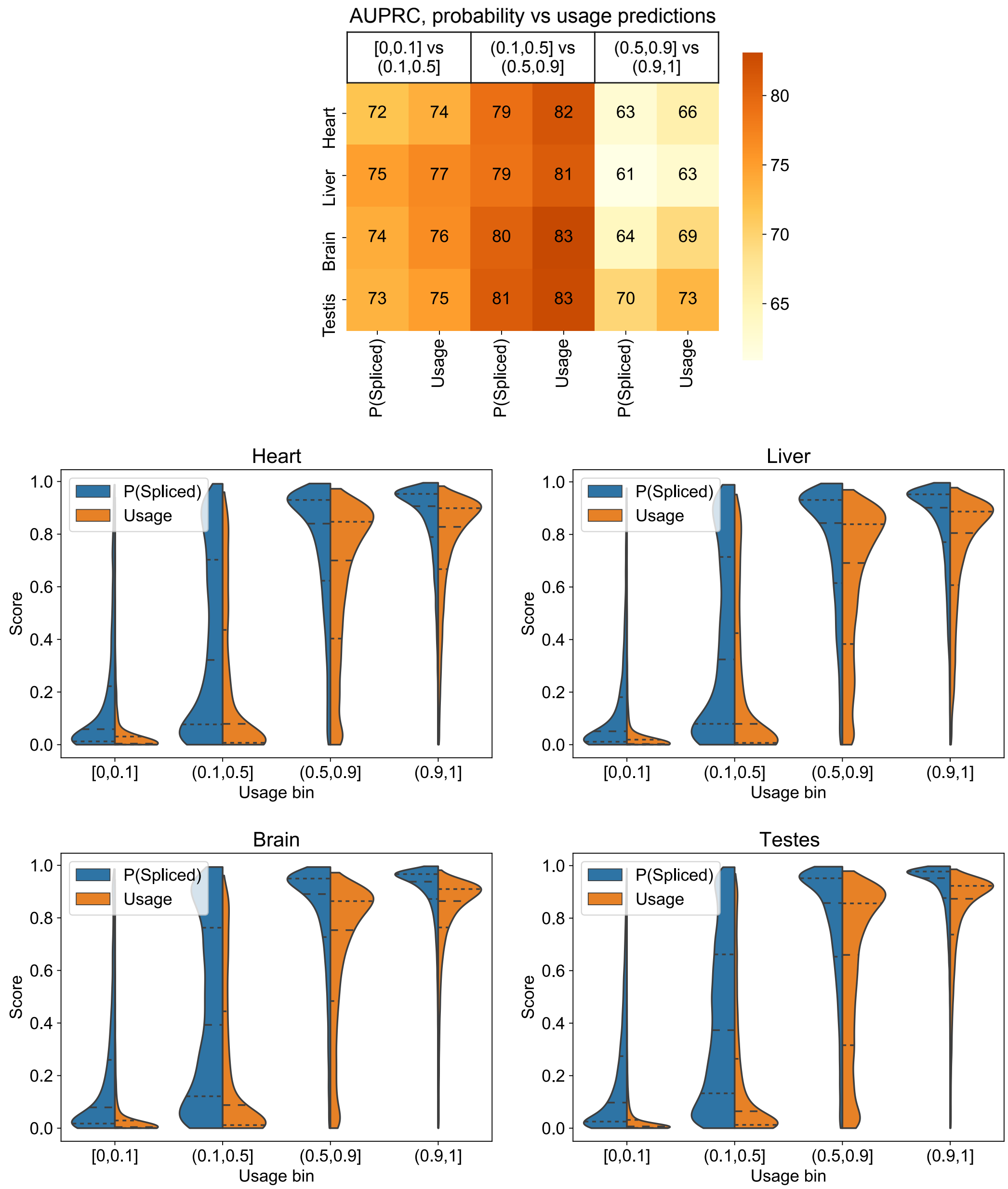

Fig. S13. Comparison of training on binary classification of splice sites versus continuous usage estimates. Heatmap shows AUPRC for classifying splice sites in different usage bins using Pangolin's predictions of the sites' usage and of P(Spliced), the probability that the sites are spliced. As expected, directly training to predict usage improved performance as compared to using the probability that a dinucleotide is a splice site. Violin plots show the distribution of Pangolin's scores (probability and usage predictions) for sites estimated to be used at different ratios empirically in heart, liver, brain, and testis.

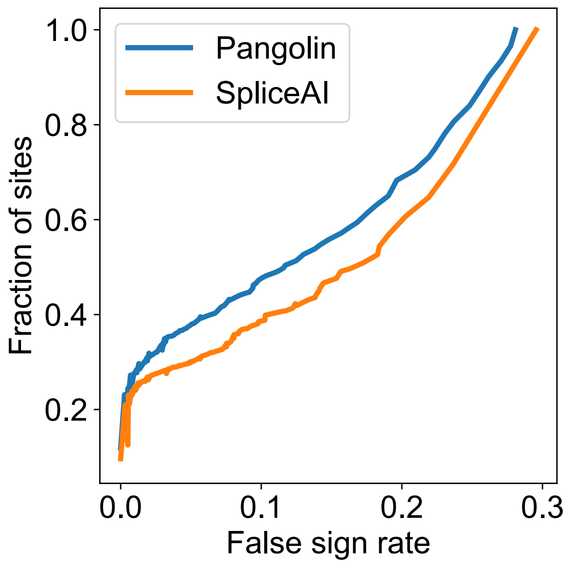

Fig. S14. Comparison between Pangolin and SpliceAI for predicting inter-species variation in splice site usage. Fraction of sites with large differences in splicing ($|\Delta \text{usage}| \geq 0.5$) between chimp and human (1,560 total sites) for which predictions were made at different false sign rates.

	Top-1				AUPRC			
	Pangolin, mid sim	SpliceAI, mid sim	Pangolin, low sim	SpliceAI, low sim	Pangolin, mid sim	SpliceAI, mid sim	Pangolin, low sim	SpliceAI, low sim
Heart	82	80	80	78	87	80	85	78
Liver	76	73	74	70	82	72	79	68
Brain	79	77	75	73	85	78	81	73
Testis	75	70	73	67	82	75	79	72

Table S3. Evaluations on subsets of test genes. Table displaying top-1 scores and AUPRC for Pangolin and SpliceAI on the test chromosomes after filtering genes that show moderate (or higher) homology (mid sim) and genes that show weak (or higher) homology (low sim) to rat, mouse, and rhesus macaque genes in the training set.

	Human, merged, -usage	Multi, merged, -usage	Multi, merged	Multi
Heart (1)	0.808	0.832	0.855	0.871
Heart (2)	0.807	0.831	0.856	0.871
Liver (1)	0.706	0.733	0.773	0.812
Liver (2)	0.703	0.731	0.774	0.812
Brain (1)	0.774	0.804	0.828	0.845
Brain (2)	0.774	0.805	0.827	0.845
Testis (1)	0.77	0.791	0.796	0.795
Testis (2)	0.77	0.79	0.797	0.795

Table S4. Comparison of AUPRC between models trained on multiple species and on human only. Table showing comparison of AUPRC computed over the test set for models trained on human, rhesus macaque, mouse, and rat RNA-seq data to models trained on human data only. Models trained on multiple species show consistently better performance.
