# Supplementary material for: Predicting RNA splicing from DNA sequence using Pangolin
Source: Genome Biol. 2022 Apr 21;23:103. doi: 10.1186/s13059-022-02664-4 (PMC9022248; doi:10.1186/s13059-022-02664-4)
Supplement: Supplementary file 3 — Additional file 3 Review history. [file 13059_2022_2664_MOESM3_ESM.docx]

**1^st^ round**

**Reviewer 1**

Possibly the most interesting feature of Pangolin is the tissue specific expression. Although the authors report the results, they have not provided any explanation of what distinguishes (for example) brain specific splicing from liver specific splicing. Is there any clear signal?

The authors claim that orthology and paralogy was removed from the training set by using the relationships in BioMart. But these cross-species relationships are one-to-one only, so duplications in the last 100 million years or so in rats/mice (which lets face it will be common) are not eliminated, and nor are the rat/mouse/macaque duplications of genes in the training set excluded.

In general the paper is clear, but the methods section could do with a bit more detail. It feels as if the authors wrote this part in a hurry.

Minor details

The authors claim that Pangolin works better at predicting pathogenic variants that affect canonical splice sites. However, this information is NOT in the MC column of ClinVar. I assume that the authors meant to say "annotated" splice sites.

Are the blocks of 15,000 bases as the methods claim? According to the figures the blocks should be 10,000 bases.

"that there exist prevalent mutational effects"

--> "that prevalent mutational effects exist"

CDF not defined (in the method section)

**Reviewer 2**

In this manuscript, Zeng and Li presented a deep learning method Pangolin to predict tissue-specific splicing from primary sequences. The general approach is somewhat similar to the recently published work SpliceAI, but this study has several extensions in model architecture and training: 1. Pangolin predicts usage of splice sites in four discretized bins instead of a binary state. 2. The output layer has output of splicing in multiple tissues (heart, liver, brain, and testis; it should be pointed out that SpliceAI did something similar). 3. The model was trained using data from several species. The performance of the model was evaluated in several prediction tasks and improvement over SpliceAI and several other methods was observed. These results are relatively incremental but nevertheless represent important efforts for an important problem of high significance. Potential utility of such methods was nicely demonstrated and the manuscript was very well written.

Main points:

1. Compared to SpliceAI, Pangolin adopted several changes in both network architecture and training data. It is not clear which contributed to the improvement of Pangolin over SpliceAI. It will be very helpful to train several intermediate models to demonstrate the point, e.g., 1) SpliceAI model with expanded dataset from multiple species; 2) intermediate model 1 + splice usage prediction in single tissue; 3) SpliceAI model with multiple tissue output.

2. SpliceAI achieved high accurate in distinguishing exons with high vs low inclusion, but the performance is moderate for exons with intermediate levels. How about Pangolin? It will be informative to plot the measured vs predicted PSI values in both training and testing datasets.

3. There are multiple datasets that measured a large number of genetic variation or mutations on splicing, including MaPSy, VEX-seq, and MFASS, while only the MFASS dataset was used for evaluation. It will be helpful to compare different methods on the other datasets to provide a more comprehensive view of the performance of each method.

4. By looking at the mutations predicted to affect splicing, most appears to be either in the splice sites or very proximal to the splice sites. It will be important to check the impact of relatively distant mutations, and how Pangolin performs and compares to other methods.

Minor points:

1. The authors claim they are predicting "continuous" splicing usage, but they are really only predicting discretized bins. I suggest the authors made this clear to avoid confusion.

2. When the authors include data from other species for model training, they excluded any genes with orthology to test genes. Any change false negative in detecting orthologous genes can lead to information leaking? I guess a potentially more stringent way is to only include genes that are orthologous to human genes in the training dataset.

3. Nowadays, RNA-seq data are available for many more tissues than the four used for model evaluation. Why these four are chosen? It is not completely convincing that they represent major organs, while others are not. It will be helpful to include analysis of a more comprehensive panel of tissues.

**Authors’ response**

Reviewer 1

Possibly the most interesting feature of Pangolin is the tissue specific expression. Although the authors report the results, they have not provided any explanation of what distinguishes (for example) brain specific splicing from liver specific splicing. Is there any clear signal?

Thank you for this suggestion. To characterize what Pangolin has learned to predict tissue-specific splicing, we used a feature-attribution method called DeepLIFT. For a given output, DeepLIFT assigns an importance score to each base in the input, where a high importance score indicates that a base contributes significantly to the output. We ran DeepLIFT on brain- and testis-specific sites that Pangolin was able to predict, as there were many more such sites compared to heart- and liver-specific sites (663 and 1,738 vs 60 and 46 sites). We considered a tissue-specific site to be correctly predicted if the measured and predicted differences in usage from the mean across tissues were both >0.05, and used a vector of all zeros as the reference input for DeepLIFT. Additionally, for both brain and testis, we ran DeepLIFT on five random sets of non-tissue-specific sites with similar usage distributions as the tissue-specific sites; these sites served as our controls.

We identified clusters of high-importance bases by calling peaks with the peak caller MACS, using an absolute importance score cutoff of 0.001, minimum peak size of 5, and maximum allowed gap size of 10 as parameters. Although MACS is generally used for finding peaks in genomics data (e.g. ChIP-seq, or ATAC-seq), visual inspection of the peaks identified using this approach on DeepLIFT scores appeared to reflect well high-scoring regions that we would annotate by hand.

After extending each peak by 5 bases on either side, we conducted a differential enrichment analysis on the sequences of the peaks to identify motifs enriched in brainand testis-specific sites relative to the control sites. To do this, we used the method Multiple Em for Motif Elicitation (MEME), allowing for any number of motif repetitions to occur in each input sequence as a parameter. We calculated the differential enrichment of motifs in tissue-specific sequences against all sets of control sequences and conducted an equal number of comparisons of control sequences against control sequences.

At an E-value cutoff of 0.01, we found no significant motifs when comparing control against control sequences, but five differentially enriched motifs in brain and six in testis for tissue-specific sites. Next, we characterized the top five differentially enriched motifs from each tissue. We first noticed that the motifs for both brain and testis look roughly similar (motif sequence logos are shown in Supplementary Figure 5, reproduced below). To analyze this further, we found that the mean pairwise correlation between the brain motifs was 0.42, compared to 0.53 for the testis motifs; however, the mean pairwise correlation of the brain motifs to the testis motifs was 0.48, indicating that the brain and testis motifs are not specific to their respective tissues.

To directly test for differences between brain and testis motifs, we again used MEME to identify differential enrichment of motifs in testis sequences relative to brain sequences and vice versa. We did this for brain- and testis- specific sites, and for our control sites (testing brain against testis control sites and vice versa). While one motif was significantly enriched in testis-specific sites relative to brain-specific sites, there was also one motif significantly enriched in a set of testis control sites relative to a set of brain control sites. Therefore, it is unclear whether Pangolin has learned sequence features that distinguish testis-specific sites from brain-specific sites, or broader differences in splicing between the two tissues.

Next, we wanted to look at the positional distributions of the top five differentially enriched motifs from each tissue. Using FIMO, a tool from the MEME Suite, we scanned the motif across 1,000 bases surrounding each tissue-splice site, and plotted histograms of the locations of the significant motif hits (see Supplementary Fig. 5 for examples, reproduced below). While significant hits are unevenly distributed across the entire range of positions, they are generally low in number near the splice site. For many pairs of motifs, the distributions are roughly similar, indicating that these motifs capture similar sequence features (Supplementary Fig. 5).

Finally, we tested more stringent cutoffs for identifying motifs. First, we identified sharper peaks by running MACS using an absolute importance score cutoff of 0.005 and maximum allowed gap size of 2. Second, instead of using MACS, we identified peaks using a sliding-window approach, where we computed the average importance scores over four bp windows, kept windows with average scores >=0.005, and merged overlapping windows---we called the resulting windows to be peaks. For both approaches, we searched for differential enrichment of motifs but over a tighter motif size range than before (8-15 bp using STREME from the MEME Suite). In comparing testis-specific sites to sets of control sites, we found one motif at an E-value cutoff of 0.05 using each approach (Supplementary Fig. 5). We found no other motifs, neither for the control against control nor in the brain-specific against control comparisons. Overall, we have identified motifs enriched in brain- and testis- specific splice sites relative to non-tissue-specific splice sites, but these motifs are not themselves tissuespecific. More sensitive analyses may be needed to detect further tissue-specific motifs.

We have now added a Supplementary Note to present these results, and added a sentence in the main text to point to this analysis:

*Additionally, we find that there is highly significant differential enrichment of multiple sequence motifs near correctly-predicted brain- and testis-specific splice sites relative to non-tissue-specific splice sites; though, to the best of our knowledge, the motifs identified do not correspond to the binding motif of any well-studied splice factor (Supplementary Fig. 5, Supplementary Note 5)*

The authors claim that orthology and paralogy was removed from the training set by using the relationships in BioMart. But these cross-species relationships are one-to-one only, so duplications in the last 100 million years or so in rats/mice (which lets face it will be common) are not eliminated, and nor are the rat/mouse/macaque duplications of genes in the training set excluded.

Thank you for this comment. To more stringently filter test-set genes with high similarity to training-set genes, we used Liftoff, a genome annotation lift-over tool, to map human genes to the mouse, rat, and rhesus macaque genomes. Liftoff uses Minimap2 to align gene sequences to the target genome, finding the alignment of exons that maximizes sequence identity while preserving gene structure, and is able to identify copies of genes. We applied two cutoffs to the alignment coverage and sequence identity in the exons/CDS to filter genes from the test set. Using a cutoff of 0.4 for both alignment coverage and sequence identity, 29% of genes in our original test set were retained (3,037 genes), and using a cutoff of 0.2, 15% of genes were retained for testing (1,585 genes).

The top-1 accuracies and AUPRC for Pangolin and SpliceAI on the more stringent test sets (Supplementary Table 3, replicated below; mid sim and low sim correspond to the 0.4 and 0.2 cutoff respectively). The improvements of Pangolin over SpliceAI are similar for the original, mid sim, and low sim test sets: 5.2%, 4.1%, and 5.0% increases in top-1 accuracy, and 10.6%, 10.2%, and 11.5% increases in AUPRC respectively. These results indicate that Pangolin’s improvements do not come from memorization of orthologous sequences from the training data. We note that there is a slight decrease in Top-1 and AUPRC scores from mid sim to low sim test set, the decrease is very modest and may simply reflect that the genes in the test set are very different from those in the training set both in terms of sequence, and also in terms of splicing.

We have now added a Supplementary Note to present these results, and added a sentence in the main text to point to this analysis:

*In addition, we found that the improvements in Pangolin's performance were similar for genes with low identity to training-set genes from other species, indicating that any orthologous sequence that were not filtered out from our training set only minimally affects Pangolin's predictions (Supplementary Note 2)*

In general the paper is clear, but the methods section could do with a bit more detail. It feels as if the authors wrote this part in a hurry.

Thank you for this comment, we have now added a substantial amount of details to the methods section and carefully edited it. We hope that our effort will be obvious in the revised manuscript.

Minor details

The authors claim that Pangolin works better at predicting pathogenic variants that affect canonical splice sites. However, this information is NOT in the MC column of ClinVar. I assume that the authors meant to say "annotated" splice sites.

We are unsure about the version of the file you are referring to. Our ClinVar VCF has MC column information about splice sites: for example, “MC=SO:0001575|splice_donor_variant”. We think that perhaps our ClinVar file differs. That being said, we have now replaced mentions of “canonical splice variants” to “variants in annotated splice sites”.

Are the blocks of 15,000 bases as the methods claim? According to the figures the blocks should be 10,000 bases.

Sorry for the confusion. Pangolin was trained using 15,000 bases with 5,000 outputs for efficiency. The sequence context for each base is 10,000 bases. We have revamped our methods section to add a substantial amount of detail.

"that there exist prevalent mutational effects" --> "that prevalent mutational effects exist"

We have now updated this.

CDF not defined (in the method section)

Many thanks for noticing this. We have now defined this.

Reviewer 2

In this manuscript, Zeng and Li presented a deep learning method Pangolin to predict tissue-specific splicing from primary sequences. The general approach is somewhat similar to the recently published work SpliceAI, but this study has several extensions in model architecture and training: 1. Pangolin predicts usage of splice sites in four discretized bins instead of a binary state. 2. The output layer has output of splicing in multiple tissues (heart, liver, brain, and testis; it should be pointed out that SpliceAI did something similar). 3. The model was trained using data from several species. The performance of the model was evaluated in several prediction tasks and improvement over SpliceAI and several other methods was observed. These results are relatively incremental but nevertheless represent important efforts for an important problem of high significance. Potential utility of such methods was nicely demonstrated and the manuscript was very well written.

Thank you for your interest in our work and for your kind comments, we carefully considered your suggestions and believe to have addressed them in a thorough manner. We hope that you will agree with us!

Main points:

1. Compared to SpliceAI, Pangolin adopted several changes in both network architecture and training data. It is not clear which contributed to the improvement of Pangolin over SpliceAI. It will be very helpful to train several intermediate models to demonstrate the point, e.g., 1) SpliceAI model with expanded dataset from multiple species; 2) intermediate model 1 + splice usage prediction in single tissue; 3) SpliceAI model with multiple tissue output.

Thank you for this comment. We now report results for four “intermediate” models: 1. A model trained on human data only, with prediction on merged tissues with no usage labels---only binary labels of spliced or unspliced. We merged tissues by taking the union of splice sites across tissues. This model is most similar to that of SpliceAI, which was trained to predict binary labels in a tissue-agnostic manner.

2. A model trained on data from all species, with prediction on merged tissues with no usage labels.

3. A model trained on data from all species, with prediction on merged tissues and with usage labels. We trained this model to predict both the existence and usage of splice sites. We merged per-tissue usage labels by taking the average across tissues for which usage labels were available.

4. A model trained on data from all species, with separate predictions for each tissue and with usage labels. This model is equivalent to the full Pangolin model, except we train it without fine-tuning on each individual tissue to allow for comparisons to models 1-3.

For each model, we trained until the validation loss stopped decreasing, which took 14 epochs for model 1 and 6 epochs for the other models, following the training procedure described in Methods except without the tissue-specific fine-tuning step. We repeated the training procedure twice, and found that there was minimal variance between the replicates (Supplementary Table 4, reproduced below). Furthermore, as expected, we found that model 1 produces similar results as SpliceAI (average AUPRC of 0.764 for model 1, AUPRC of 0.765 for SpliceAI). Training on data from multiple species improved predictions (AUPRC increase of 2.6%, model 2 over model 1), as did training with usage labels (AUPRC increase of 2.4%, model 3 over model 2) and having separate output heads for each tissue (AURPC increase of 1.8%, model 4 over model 3). These results demonstrate that each of the changes we made to Pangolin's models (compared to SpliceAI's models) led to consistent improvements in predictive performance.

We have now added a Supplementary Note to present these results, and added the following sentences in the main text to point to this analysis:

*To better understand the improved performance of Pangolin over SpliceAI, we trained multiple intermediate Pangolin models and evaluated improvements of AUPRC separately for each model. We found that (i) using splicing data from multiple tissues instead of just one, (ii) using data from multiple species instead of just human, and (iii) using quantitative measurements of splice site usage instead of binary classifications, all improved AUPRC (Supplementary Note 3). These observations indicate that increasing the number of species and tissues used for training can further improve our model's performance*

2. SpliceAI achieved high accurate in distinguishing exons with high vs low inclusion, but the performance is moderate for exons with intermediate levels. How about Pangolin? It will be informative to plot the measured vs predicted PSI values in both training and testing datasets.

Thank you for this interesting question. To answer it, we plotted the distributions of the predicted usage values for the test set (in blue) and the training set (in orange) for different bins of intermediate splice usage: 0.1 to 0.3, 0.3 to 0.5, 0.5 to 0.7, and 0.7 to 0.9. We note clear separation of the predicted usage values between the lowest and highest bins, but the distributions of scores for sites with usage between 0.3 and 0.5, and between 0.5 and 0.7 are more similar and also more dispersed. Therefore, Pangolin is unable to clearly distinguish between sites with different levels of intermediate usage. This is true for both the training and test datasets. However, for all tissues, the median score in each bin is in between that of the immediately lower and higher bins, indicating that Pangolin has learned some differences between sites of intermediate usage. Thus, we believe that Pangolin’s performance on accurately predicting the usage of “moderately” used exons does not significantly improve on that of SpliceAI. Consequently, we decided not to include this analysis in the revised version of our manuscript.

3. There are multiple datasets that measured a large number of genetic variation or mutations on splicing, including MaPSy, VEX-seq, and MFASS, while only the MFASS dataset was used for evaluation. It will be helpful to compare different methods on the other datasets to provide a more comprehensive view of the performance of each method.

Thanks for this suggestion. We further compared the performance of Pangolin, SpliceAI, and MMSplice on the MaPSy dataset, obtained from the MMSplice repository:

https://github.com/gagneurlab/MMSplice_paper. The MaPSy dataset provides measures of the splicing efficiency, the proportion of spliced RNAs in the set of total RNAs, for thousands of variants in both in vitro and in vivo assays. For Pangolin and SpliceAI, we calculated the Pearson correlation between the change in splice score for each method and the measured change in splicing efficiency in vitro and in vivo (defined as log2((mo/mi)/(wo/wi)), where mo, wo are the mutant and wildtype spliced RNA read count, and mi, wi are the mutant and wildtype unspliced RNA read count respectively). We computed scores for all sites for which this log ratio could be calculated. For MMSplice, we report the Pearson correlations given in the MMSplice paper, which were calculated for a subset of sites (since MMSplice was trained on the remaining sites). Pangolin achieved correlations of 0.51 and 0.61 for the in vitro and in vivo assays respectively, compared to correlations 0.40 and 0.50 for SpliceAI and 0.57 and 0.37 for MMSplice. While MMSplice performs the best on the in vitro assay, Pangolin achieves the highest correlation on the in vivo assay. We plot Pangolin’s results in Supplementary Figure 2 (reproduced below).

We added a sentence and a supplementary figure on this analysis in the revised version of the manuscript:

*Further benchmarking of Pangolin, SpliceAI, and MMSplice on in vivo splicing efficiency data from a massively parallel splicing assay (MAPSy), revealed that Pangolin predictions of genetic effects were most highly correlated with that measured using MAPSy (Pearson correlations of 0.61, 0.50, 0.37 for Pangolin, SpliceAI, and MMSplice respectively) (Supplementary Fig. 2, Methods).*

4. By looking at the mutations predicted to affect splicing, most appears to be either in the splice sites or very proximal to the splice sites. It will be important to check the impact of relatively distant mutations, and how Pangolin performs and compares to other methods.

Thank you for this interesting question. To understand Pangolin’s performance on mutations that are relatively far from the splice sites, we further analyzed the MFASS dataset. We report Pangolin’s performance compared to SpliceAI for variants that are 0- 9, 10-19, 20-29, 30-39, and 40-53 bases away from the nearest splice site of the exon. Pangolin’s performance is high for variants close to the splice sites (AUPRC of 0.75, 0-9 bases away), but low to moderate for variants farther away (AUPRC <= 0.34). This may be explained by the substantially fewer number of splice disrupting variants (SDVs) in the more distant bins (8.8% SDVs, 0-9 bases away, vs <=2.9% SDVs in the other bins). However, in all cases, Pangolin outperforms SpliceAI (average increase in AUPRC of 0.10 across all bins). We plot the precision-recall curves and report their AUPRC in Supplementary Figure 1 (reproduced below).

We added a sentence and a supplementary figure on this analysis in the revised version of the manuscript:

*Interestingly, we found that Pangolin's performance is substantially better for variants near splice sites (AUPRC of 0.75, distance of 0-9 bases) than for farther variants (AUPRC <0.35, distance >9 bases), which may be attributable to the relative rarity of distant splice-disrupting variants (Supplementary Fig. 1).*

Minor points:

1. The authors claim they are predicting "continuous" splicing usage, but they are really only predicting discretized bins. I suggest the authors made this clear to avoid confusion.

As a clarification, the training/predictions are indeed on usage estimates, which are ratios between 0 and 1. Perhaps there was some confusion because binned usage was used as the evaluation in supplementary materials? We have now made this clearer in the methods section.

2. When the authors include data from other species for model training, they excluded any genes with orthology to test genes. Any change false negative in detecting orthologous genes can lead to information leaking? I guess a potentially more stringent way is to only include genes that are orthologous to human genes in the training dataset.

Thank you for this comment, which we believe we have addressed in our response to reviewer 1’s comment #2.

3. Nowadays, RNA-seq data are available for many more tissues than the four used for model evaluation. Why these four are chosen? It is not completely convincing that they represent major organs, while others are not. It will be helpful to include analysis of a more comprehensive panel of tissues.

Thank you for this comment. Using more tissues and more species is definitely a direction that we intend to take for a future version of Pangolin. Increasing the number of species and/or tissue would take an incredible amount of time at this stage, which we believe would not significantly change the manuscript. We chose these four tissues as representing some major organs, with brain and testis being two tissue types with a lot of alternative splicing. We have now edited the manuscript to reflect that these four tissues do not represent all major organs:

*To model splicing in a quantitative, tissue-specific manner, we developed Pangolin, a deep neural network that predicts splicing in four tissues -- heart, liver, brain, and testis – which represent some of the major mammalian organs (Figure 1a).*

**2^nd^ round**

**Reviewer 2**

The authors have reasonably addressed my previous critiques. I have no further comments on the revised manuscript.
